# dbGVOSCC: a comprehensive database of genetic variation for systems genetics research on oral squamous cell carcinoma

**DOI:** 10.3389/fonc.2025.1692732

**Published:** 2025-10-31

**Authors:** Yi Zhou, Yutao Wu, Wenjing Shi, Yingbo Zhang, Xingyun Liu, Yuxin Zhang, Chaoying Zhan, Bingyue Chen, Weidong Tian, Bairong Shen

**Affiliations:** ^1^ Department of Breast Surgery and Institute for Systems Genetics, Frontiers Science Center for Disease-Related Molecular Network, West China Hospital, Sichuan University, Chengdu, China; ^2^ State Key Laboratory of Oral Diseases & National Clinical Research Center for Oral Diseases & Engineering Research Center of Oral Translational Medicine, Ministry of Education & National Engineering Laboratory for Oral Regenerative Medicine, West China Hospital of Stomatology, Sichuan University, Chengdu, China; ^3^ 01life Institute, Shenzhen, China

**Keywords:** oral squamous cell carcinoma, dbGVOSCC database, cancer heterogeneity, genetic variations, precision oncology

## Abstract

**Introduction:**

Oral squamous cell carcinoma (OSCC) is a highly aggressive malignancy of the oral epithelium, marked by a high rate of lymph node metastasis and a profound negative impact on patients’ quality of life. Despite its severity, no routine screening program currently exists for OSCC. To address the genetic heterogeneity underlying OSCC, we have developed a database of genetic variation in oral squamous cell carcinoma (dbGVOSCC; http://www.sysbio.org.cn/dbGVOSCC/).

**Methods:**

OSCC literature (1991–2024) was queried from PubMed and screened manually and via PubTator, following predefined inclusion/exclusion criteria. Entities and relations were extracted from qualifying articles and organized into tables. The database adopted a browser/server architecture using HTML and XAMPP. Front-end was built with HTML and CSS for web display; server-side used Apache for infrastructure, MySQL for data management, and PHP/JavaScript for backend-frontend integration. Bioinformatics included mapping genes to STRING (confidence >0.9), hub gene identification via PPI degree centrality, and GO/KEGG enrichment with clusterProfiler (FDR-corrected). Usability was assessed using SUS and NPS surveys.

**Results:**

dbGVOSCC comprises 1,788 somatic genetic variation entries from 400 original studies and 106,079 clinical samples, covering epimutations/methylations (329), SNPs (411), point mutations excluding SNP (258), indels (98), CNVs (348), LOH (28), one locus mutation, plus 333 unspecified mutations. We curated 817 biomarker-linked variations (diagnostic n=71, therapeutic n=175, prognostic n=291; 277 multi-application). PPI analysis highlighted 15 key genes (e.g., TP53, CTNNB1, AKT1, EGFR, PIK3CA). Enrichment implicated proliferation, adhesion/migration, p53/DNA damage response, and PI3K–Akt signaling. User testing showed SUS 88.75 (grade A) and NPS 90.

**Discussion:**

dbGVOSCC represents a robust and reliable knowledge base, offering clinicians and researchers an open-source platform for personalized genotype-phenotype association studies and systems genetics research into the mechanisms of OSCC.

## 1 Introduction

OSCC originates from the squamous epithelial cells of the oral cavity and arises in various sites, including the gums, hard palate, tongue, buccal mucosa, lips, and adjoining structures. It represents the most common form of oral cancer, accounting for over 90% of cases globally (1). In 2022, oral cavity cancers—including OSCC—accounted for approximately 188,438 deaths worldwide and an estimated total of 380,000 new cases, as reported by GLOBOCAN (2, 3). With over 650,000 new head and neck cancer cases each year, OSCC is one of the most aggressive subtypes within this category (4).

Although the incidence and mortality of OSCC have gradually declined since the 1970s, the absence of standardized screening programs and reliable blood-based diagnostics remains a major obstacle to early detection. Consequently, cervical lymph node metastases are present in approximately 40% of patients at diagnosis (5). Moreover, the prognosis is markedly poorer for these patients, with a 5-year survival rate of only 25–40%, compared to about 90% in non-metastatic cases (6). While cetuximab, an epidermal growth factor receptor (EGFR)-targeting monoclonal antibody, has been approved for the treatment of head and neck squamous cell carcinoma since 2006 (7, 8). However, its therapeutic efficacy is influenced by the genetic profile of individual patients. Genetic mutations, particularly in driver genes such as TP53, PIK3CA, and KRAS, are critical to OSCC pathogenesis and progression (9). For example, PAIP1 promotes OSCC metastasis (10), while polymorphisms in genes related to vascular endothelial growth factor (VEGF), hypoxia-inducible factor-1α (HIF-1α), and T-cell regulation are associated with clinical outcomes (11–13). Additionally, epigenetic modifications, including amplified factors such as RUVBL1 and protein tyrosine kinase 6 (PTK6), play pivotal roles in driving cancer proliferation and influencing immunotherapy responses (14, 15).

Most OSCC patients have a history of long-term smoking or alcohol consumption, whereas cases among non-smokers and non-drinkers are relatively uncommon (16). Studies suggest that certain substances, such as Catha edulis, Cannabis sativa, and Areca catechu, exert stronger carcinogenic effects than traditional tobacco, particularly in regions such as Pakistan, India, Afghanistan, and Iran (17). Although alcohol itself is not directly carcinogenic, it acts as a solvent that enhances the penetration of carcinogens into the oral mucosa (18). Individuals who both smoke and consume alcohol heavily have been reported to face up to a 30-fold greater risk of developing OSCC compared with abstainers. Notably, genetic mutations—including FAT1, CASP8, CDKN2A, and NOTCH1—further increase susceptibility by disrupting carcinogen metabolism (9). These mutations are more frequently observed in OSCC than in other head and neck cancers, with their prevalence varying across populations exposed to different risk factors.

Complex diseases like OSCC often arise from the cumulative effects of multiple genetic variants, which can only be effectively studied through systems genetics approaches (19, 20). The emergence of the fourth scientific discovery paradigm, driven by precision medicine and data integration, underscores the need for comprehensive genomics and clinical omics data (21). A comprehensive knowledge database is essential for enabling system-level analysis of these variants and understanding the molecular mechanisms underlying OSCC. Existing databases, such as the oral cancer-related gene database OCDB v.2 (22), the Copenhagen OSCC database (23), and dbGENVOC (24), have notable limitations. For example, OCDB v.2 includes only 374 genes without verification from original studies, and its data have not been updated since 2011. The Copenhagen OSCC database lacks mutation data and was not published due to privacy concerns. While dbGENVOC provides genomic data for 325 Indian oral cancer patients and 118 patients from published literature, it lacks detailed clinical information. The Cancer Genome Atlas (TCGA) offers robust genomic data for 172 to 361 cases of lip, oral cavity, and pharyngeal cancers, but its samples are primarily from the US and Canada, limiting its global applicability (25).

To address these gaps, we developed dbGVOSCC, a comprehensive database integrating fragmented OSCC-related genetic variants with corresponding data extracted from the publications. Building upon the initial version of dbGVOSCC, we have conducted bioinformatics analyses, including protein-protein interaction network studies, to investigate these variants at a systems biology level. Biomarker information has also been curated and classified to examine key genes and their roles in OSCC. The database includes manually curated clinical and topographical data from original articles, providing users with real-world clinical insights.

## 2 Materials and methods

### 2.1 Data sources

All data for the OSCC Genetic Variation Database were curated from the public PubMed database (www.ncbi.nlm.nih.gov/PubMed). The search was conducted using the following query: (oral squamous cell carcinoma[ti] OR OSCC[ti]) NOT review[ptyp] AND English[LA] AND (1991/01/01[DP]: 2024/12/31[DP]).

This search retrieved 8,657 relevant articles published between January 1, 1991, and December 31, 2024. Abstracts of these articles were reviewed, and the literature was manually selected based on predefined inclusion and exclusion criteria.

Inclusion Criteria:

Epidemiological studies on genetic variations in OSCC published in PubMed.
Studies involving patients clinically diagnosed with OSCC and their corresponding control samples.

Exclusion Criteria:

Articles not focused on genetic variation in OSCC.
Studies with incomplete or unavailable data.

To facilitate data annotation, PubTator, an automated biomedical annotation tool, was employed to extract concepts such as genes (26) and mutations from the full text (27, 28). Articles mentioning a specific gene or mutation less than once were excluded from further analysis. Subsequently, the full texts of the remaining articles were manually reviewed, and studies with incomplete data or insufficient information were excluded.

Following this rigorous screening process, a total of 400 articles were selected as the original data source for dbGVOSCC. These articles form the foundation of the database, ensuring the inclusion of high-quality, reliable information for systems genetics studies of OSCC.

### 2.2 Database implementation

**Figure 1:**
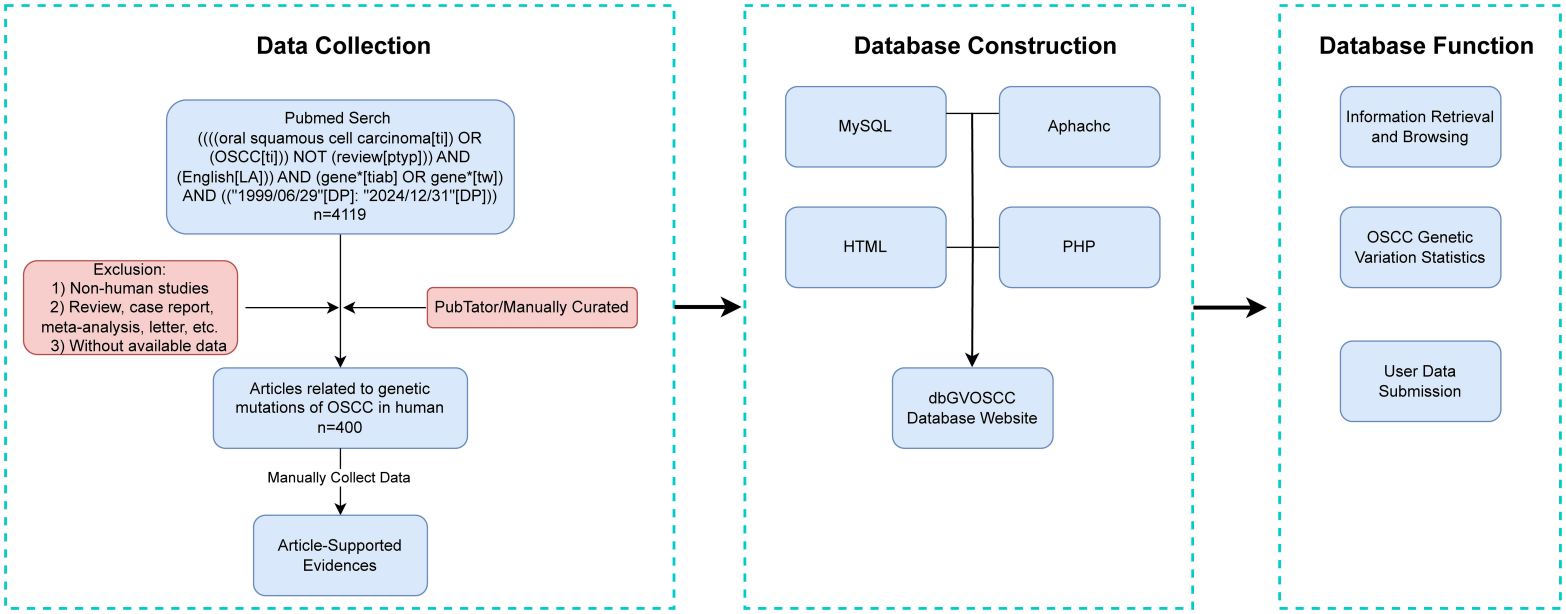
The dbGVOSCC construction pipeline.

After screening and organizing the research literature, we systematically collected and structured the relevant information. The workflow for dbGVOSCC, including data collection, construction, and functionality, is illustrated in **Figure 1**.

dbGVOSCC adopts a browser/server (B/S) architecture, enabling users to directly access data through a web browser. The front-end was developed using HTML and JavaScript, while the back-end utilizes an Apache server connected to a MySQL database via PHP. Users can explore data through categorized browsing or perform targeted searches using the online interface. A statistics page summarizes all included data, while the submission page allows users to upload new data, which is reviewed and approved by administrators before being added to the database.

### 2.3 Database structure

**Figure 2:**
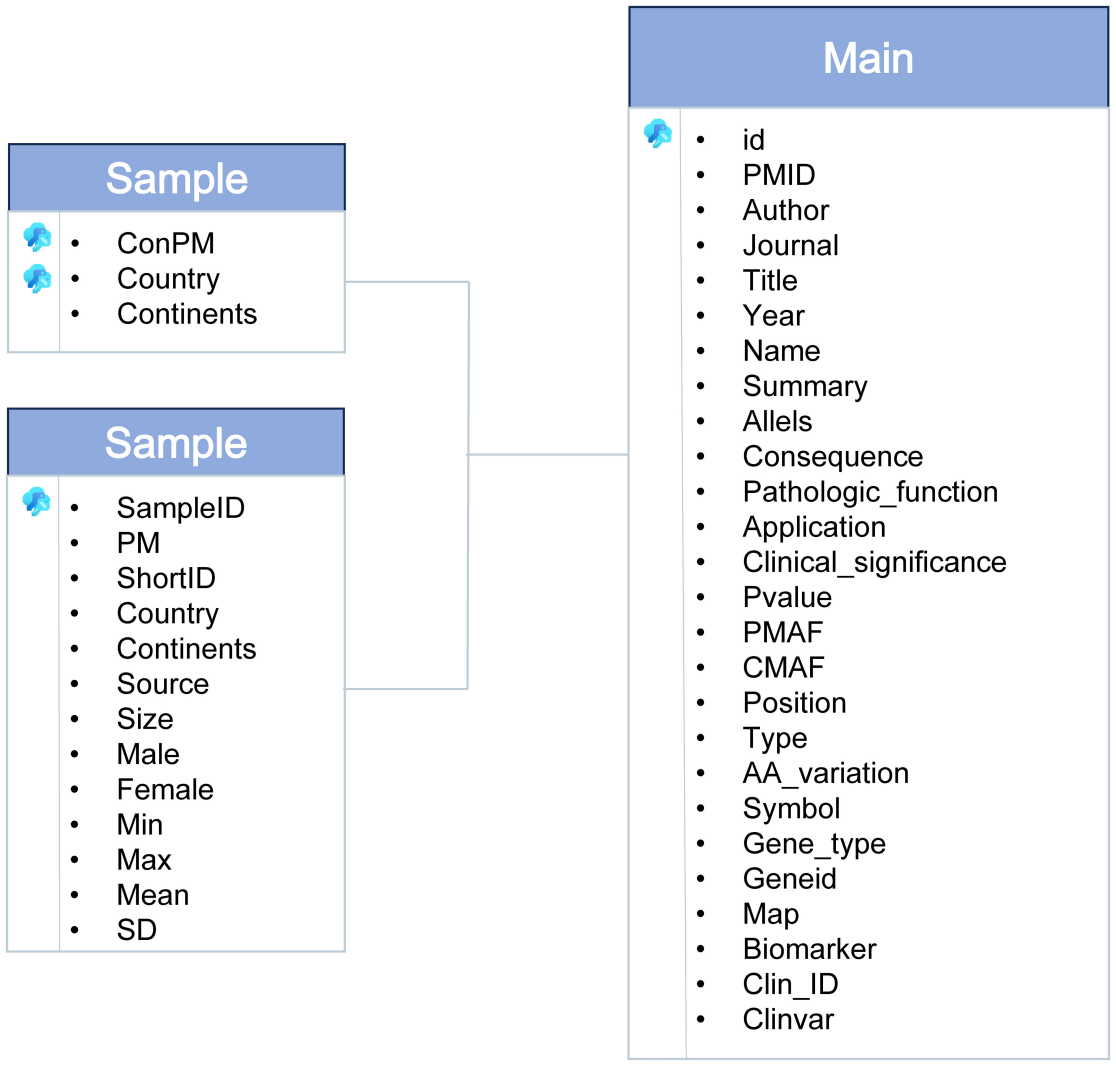
The dbGVOSCC entity-relationship (ER) diagram for the dbGVOSCC database.

The dbGVOSCC database is organized into three entity tables and one relational table, as shown in **Figure 2**. These tables encompass all data within the database:

1. Sample Table: Contains information on clinical samples, including sample ID, age, country, subject size, sex, and sample source.
2. Reference Table: Includes details of the literature, such as PMID, year, article title, authors, and journal name.
3. Mutation Table: Records mutation-related data, including main ID, pathological function, clinical significance, clinical application, patient mutation frequency, control variant frequency, and a summary.

The relational table integrates these entity tables, facilitating seamless data retrieval and analysis.

### 2.4 Bioinformatics analysis

Proteins, as the functional products of genes, play pivotal roles in metabolic and signaling pathways. To explore the associations between genetic variants and disease phenotypes, we mapped the genes from dbGVOSCC to the STRING database with a confidence score > 0.9. Using the mapped data, a protein-protein interaction (PPI) network was constructed, and node degree centrality was calculated with Cytoscape. Nodes were ranked in descending order of centrality, and the top 5% were identified as key genes.

Subsequently, functional enrichment analyses were performed using the clusterProfiler package in R (v4.2.0), with the curated OSCC‐mutated genes identified in this study as the input set. The background universe was defined as all annotated human genes available in Gene Ontology (GO) enrichment analysis and Kyoto Encyclopedia of Genes and Genomes (KEGG). Enrichment significance was assessed using a hypergeometric test with Benjamini-Hochberg false discovery rate (FDR) correction. GO enrichment covered three ontologies (biological process, molecular function, and cellular component), while pathways were annotated based on KEGG. Terms with FDR < 0.05 and fold enrichment > 2 were considered significantly enriched. For visualization, results were summarized by gene ratio (number of input genes in the term/total number of input genes), fold enrichment, point/bar size (hit gene count), and color scale (-log^10^[FDR]), and enriched terms were ranked first by FDR and then by gene ratio.

### 2.5 Validation of the efficacy and utility of dbGVOSCC

First, we performed a comparative analysis between dbGVOSCC and ChatGPT-5 to assess the effectiveness of dbGVOSCC in retrieving relevant information from scientific literature. Specifically, we submitted genetic variation data from 10 oral squamous cell carcinoma (OSCC) patients to both dbGVOSCC and ChatGPT-5. The returned results were then evaluated based on the reported relationships between genetic variations and OSCC, along with the corresponding source publications, following the evaluation criteria outlined in **Supplementary Table S1**. The comparison with ChatGPT-5 was conducted using the official GPT-5 model via https://chat.openai.com in August 2024, ensuring consistency in system version and output.

Second, we conducted System Usability Scale (SUS) and Net Promoter Score (NPS) surveys to assess the usability and user loyalty of dbGVOSCC. We collected 21 valid responses from 10 clinicians, 8 researchers, and 3 public users.

Last, we compared dbGVOSCC with the OSCC-related databases to highlight its distinct features and enhancements.

## 3 Results

### 3.1 Data statistics

Following the screening and sorting procedures described above, we identified a total of 1,788 genetic variants associated with OSCC, including 329 epimutations/methylations, 411 single nucleotide polymorphisms (SNPs), 258 point-mutations excludes SNP, 98 insertion-deletion mutations (indels), 348 copy number variations (CNVs), 28 instances of loss of heterozygosity (LOH), 1 locus mutation, and 333 unspecified gene mutations. Classified by the types of gene function mutations, including 514 protein-coding DNA genes, 21 miRNAs, and 1 pre-mRNA. The corresponding clinical data encompassed a total of 106,079 clinical samples, of which 59,440 were distinctly identified as male and 20,674 as female (**Table 1**). Clinical samples are the total sample size aggregated across all studies included in dbGVOSCC. Topographic data are the subset of those samples with the primary OSCC site explicitly annotated in the source studies, including 13, 083 samples mainly distributed in 7 different oral topographic sites (**Supplementary Figure S1**).

**Table 1:** Basic data statistics of the database.

Genetic variant type	Count
CNV	348
LOH	28
Locus mutation	1
Methylation	329
SNP	411
Point mutation excludes SNP	258
Indel mutation	98
Unspecified gene mutation	333

CNV, Copy number variation; LOH, Loss of heterozygosity; SNP, Single nucleotide polymorphism. Locus mutation: Genetic variation identified at a specific genomic locus or region, where the gene responsible for the mutation cannot be confidently determined. Unspecified gene mutation: The mutation is described in relation to OSCC, but the specific gene and/or the exact genetic mutation involved is not clearly identified.

**Figure 3:**
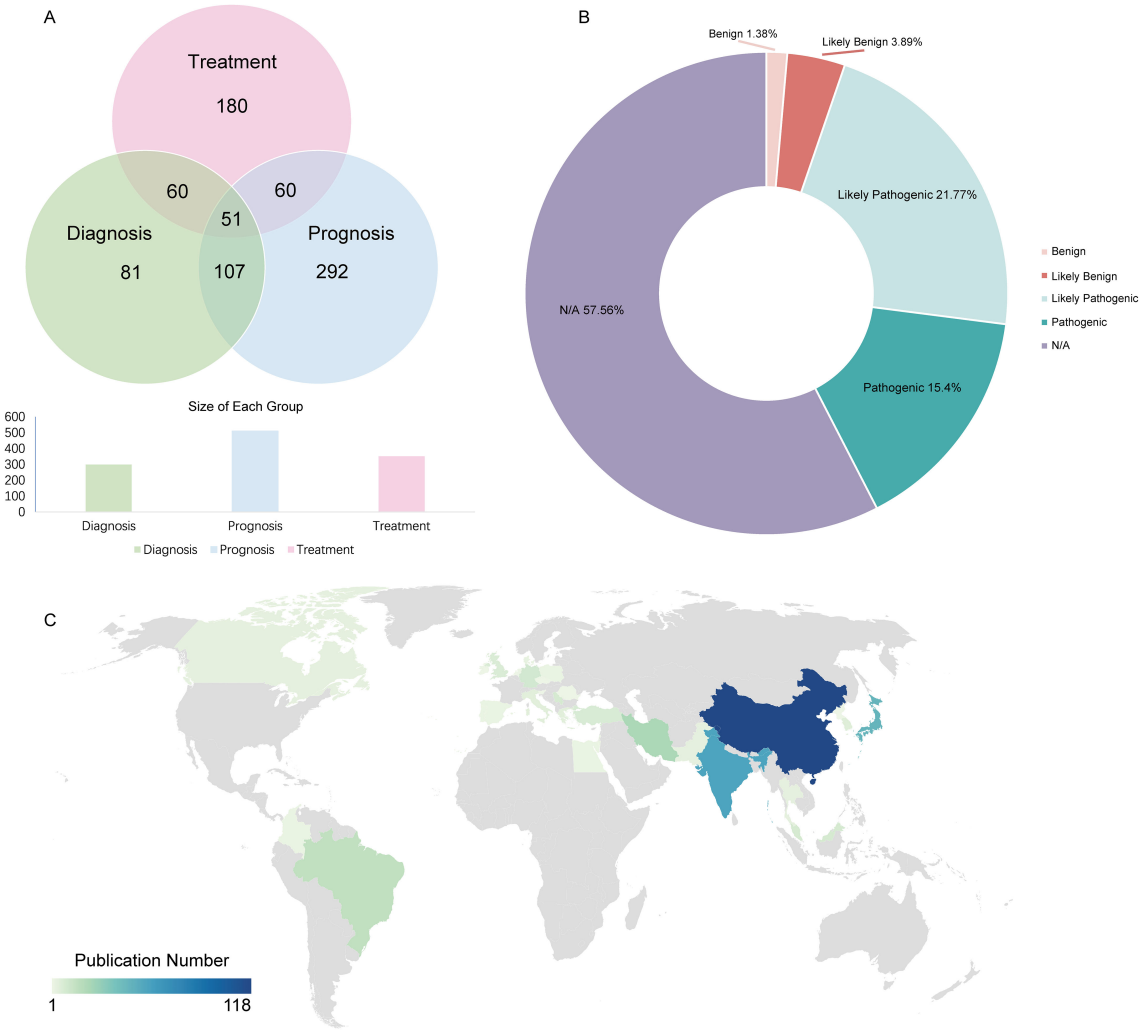
Distribution of dbGVOSCC-related biomarker types. **(A)** The distribution of biomarkers among diagnostic, therapeutic, and prognostic. **(B)** The possible consequences of corresponding gene mutations include benign, likely benign, likely pathogenic, and pathogenic responses. **(C)** The distribution of OSCC-related publications worldwide.

All 817 genetic variations were identified as potential biomarkers, with 201 explicitly labeled as such. These genetic variations were categorized into three primary application types --diagnostic, therapeutic, and prognostic --based on their roles in standard biological processes, pathological mechanisms, or pharmacological responses to therapeutic interventions (71, 175, and 291 variations, respectively) (**Figure 3A**). The distribution of variant clinical significance (benign, likely benign, likely pathogenic, pathogenic) is shown in **Figure 3B**. Among the 817 genetic variations, 277 were classified as multi-application biomarkers, meaning they were associated with two or more application types, accounting for 33.9% of the total. Based on data from 400 original research articles (**Figure 3C**), China has published the largest number of OSCC-related studies.

### 3.2 Website

**Figure 4:**
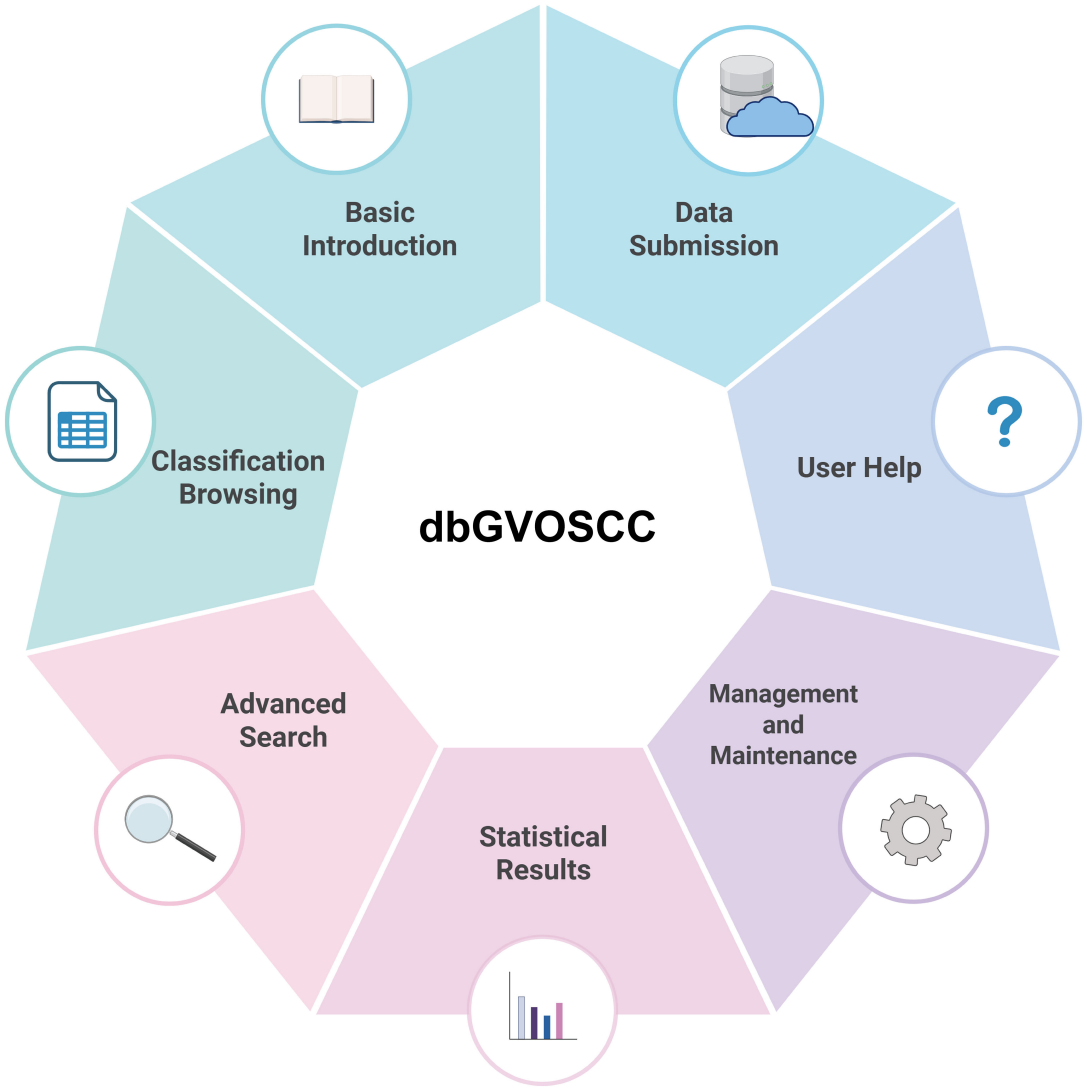
The dbGVOSCC function modules.

Based on the structure and application requirements of dbGVOSCC, we developed seven functional modules for online use, as illustrated in **Figure 4**:

1. Home page: This page provides a comprehensive overview of dbGVOSCC, including references, related information, and resources. It also includes links to associated databases and research institution websites.
2. Browse page: This page allows users to explore data through three classification methods: variation type, clinical sample source, and mutation-related gene classification.
3. Search page: This page enables users to search for specific information using mutation name, data source (location), or gene name as search criteria.
4. Statistics page: This page displays key insights, such as the distribution of mutation types, the top ten genes ranked by frequency, the chromosomal distribution of mutations, and the geographic distribution of clinical sample sources.
5. Submit page: Users can contribute new data by providing four required items: mutation name, reference, contact email, and details. Mutation name and email are mandatory fields. Submitted data is reviewed, and upon approval, it is promptly added to dbGVOSCC.
6. Help page: Offers detailed guidance on navigating and using dbGVOSCC, ensuring that users can efficiently locate the desired information.

### 3.3 Bioinformatics analysis result

**Figure 5:**
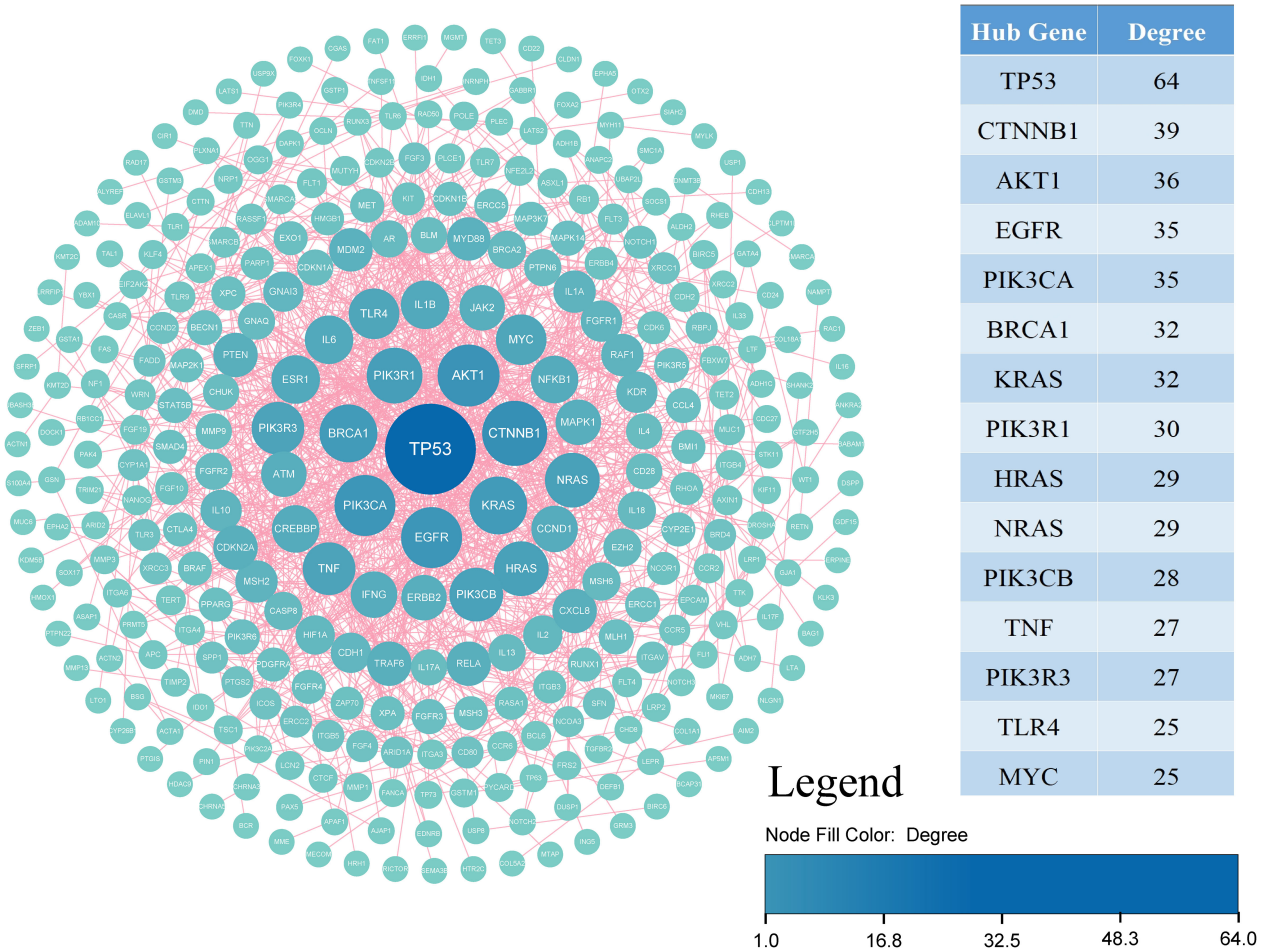
PPI network composed of OSCC-related genes.

Using STRING, identifying 283 nodes and 954 interactions. Through degree centrality analysis, we pinpointed 15 key genes—TP53, CTNNB1, AKT1, EGFR, PIK3CA, BRCA1, KRAS, PIK3R1, HRAS, NRAS, PIK3CB, TNF, PIK3R3, TLR4, and MYC—as shown in **Figure 5** and **Table 2**.

**Table 2:** Functions of key genes in OSCC.

Gene	Functions	PMID
TP53	DNA damage response, cell cycle arrest, apoptosis	29489434
CTNNB1	Wnt/β-catenin signaling; cell adhesion and transcription regulation	33531646
AKT1	Regulation of cell proliferation and growth, and participation in apoptosis and glucose metabolism	27889930
EGFR	Cell proliferation, angiogenesis, tumor invasion, metastasis, and apoptosis	33680380
PIK3CA	Tumor growth	31516747
BRCA1	DNA repairment	29896272
KRAS	Regulation of cell growth and activation	20813562
PIK3R1	Cell adhesion and development	32115621
HRAS	Transduce signals that instruct cells to grow and divide	31845386
NRAS	Transduction of signals that instruct cell growth and division	33680380
PIK3CB	Cell growth, proliferation, differentiation, and survival	32115621
TNF	Promotion of inflammation	37505766
PIK3R3	Promotion of cell proliferation, immortalization, dedifferentiation, and transformation	32115621
TLR4	NF-κB/MAPK activation, immune escape, chemoresistance	38593176
MYC	Transcription factor; proliferation, apoptosis, tumor progression	35890188

TP53, Tumor Protein P53; CTNNB1, Catenin Beta 1; AKT1, AKT Serine/Threonine Kinase 1; EGFR, Epidermal Growth Factor Receptor; PIK3CA, Phosphatidylinositol-4,5-Bisphosphate 3-Kinase Catalytic Subunit Alpha; BRCA1, Breast Cancer 1, DNA Repair Associated; KRAS, KRAS Proto-Oncogene, GTPase; PIK3R1, Phosphoinositide-3-Kinase Regulatory Subunit 1; HRAS, HRAS Proto-Oncogene, GTPase; NRAS, NRAS Proto-Oncogene, GTPase; PIK3CB, Phosphatidylinositol-4,5-Bisphosphate 3-Kinase Catalytic Subunit Beta; TNF, Tumor Necrosis Factor; PIK3R3, Phosphoinositide-3-Kinase Regulatory Subunit 3; TLR4, Toll-Like Receptor 4; MYC: MYC Proto-Oncogene, BHLH Transcription Factor.

All included genes underwent Gene Ontology (GO) and Kyoto Encyclopedia of Genes and Genomes (KEGG) pathway enrichment analyses. The top ten results in each of the four GO categories were ranked in descending order based on the number of associated genes, as detailed in **Supplementary Figure S2**.

#### 3.3.1 Gene Ontology biological process enrichment analysis

The Gene Ontology (GO) biological process enrichment analysis revealed significant involvement of genes in several key biological processes associated with oral squamous cell carcinoma (OSCC). As illustrated in **Supplementary Figure S2A**, epithelial cell proliferation, regulation of epithelial cell proliferation, muscle cell proliferation, regulation of smooth muscle cell proliferation, smooth muscle cell proliferation, gland development, and positive regulation of kinase activity indicate active growth and renewal of epithelial cells, often linked to tissue development or tumor progression. Other processes, ameboidal-type cell migration, tissue migration, and regulation of leukocyte differentiation, indicate dynamic cell motility processes, important for development, immune responses, and metastasis. These biological processes emphasize cell proliferation, tissue/organ development, migration, and immune regulation, with a strong focus on epithelial and muscle cells. The enrichment highlights pathways that underlie growth, differentiation, motility, and kinase-driven signaling, processes that are central to both normal tissue remodeling and pathological conditions such as cancer progression.

#### 3.3.2 Gene Ontology cellular components enrichment analysis

The cellular component enrichment analysis identified several key structures associated with oral squamous cell carcinoma (OSCC), as shown in **Supplementary Figure S2B**. cell-substrate junction, focal adhesion, protein complex, cell leading edge, and lamellipodium are involved in cell adhesion and migration, regulating adhesion, migration, and downstream signaling. Extrinsic component of membrane, extrinsic component of plasma membrane, and glutamatergic synapse are primarily linked to the cell surface, typically involved in signal reception and molecular recognition. In addition, transferase complex, transferring phosphorus-containing groups, and phosphatidylinositol-3-kinase (PI3K) complex represent phosphorylation-related enzymatic activity, highlighting enriched protein phosphorylation regulation. These enriched components mainly converge on cell adhesion/migration (linked to invasion and metastasis), membrane-associated signaling complexes, and kinase-driven signal transduction (such as PI3K). This suggests significant involvement in cell motility, receptor-mediated signaling, and phosphorylation regulation, processes highly relevant to tumor progression and abnormal signaling activation.

#### 3.3.3 Gene Ontology molecular function enrichment analysis

The molecular function enrichment analysis identified significant roles of key molecular activities in oral squamous cell carcinoma (OSCC), as shown in **Supplementary Figure S2C**. The most prominently enriched functions included DNA-binding transcription factor binding, RNA polymerase II-specific DNA-binding transcription factor binding, catalytic activity, acting on DNA, damaged DNA binding, and p53 binding, indicating roles in DNA metabolism, repair, and stress-response pathways, particularly involving the tumor suppressor p53. Cytokine receptor binding, growth factor binding, protein tyrosine kinase activity, transmembrane receptor protein kinase activity, and transmembrane receptor protein tyrosine kinase activity highlight communication between cells via cytokines and growth factors, critical in immune responses, proliferation, migration and differentiation. The enriched functions center on DNA interaction and repair (including p53-mediated processes) and kinase-mediated signal transduction (via growth factor and cytokine receptors). Together, these functions suggest strong involvement in gene regulation, DNA damage response, and growth/survival signaling, processes that are pivotal in both normal physiology and cancer progression. These results provide a detailed understanding of the molecular mechanisms underlying OSCC, highlighting potential targets for therapeutic intervention.

#### 3.3.4 KEGG pathway enrichment analysis

The KEGG pathway enrichment analysis highlighted several critical pathways associated with oral squamous cell carcinoma (OSCC), as shown in **Supplementary Figure S2D**. The most significantly enriched pathways included PI3K-Akt signaling pathway, MicroRNAs in cancer, and proteoglycans in cancer, which are well-known for their roles in tumorigenesis, cellular proliferation, and survival. Other notable enriched pathways involved specific cancer types, including breast cancer, prostate cancer, bladder cancer, and melanoma, reflecting the shared molecular mechanisms between OSCC and these malignancies. Chronic myeloid leukemia, platinum drug resistance, and Central carbon metabolism in cancer pathways were also enriched, indicating potential links to treatment resistance and therapeutic targeting. These findings provide valuable insights into the molecular mechanisms driving OSCC and highlight key pathways for future therapeutic interventions.

### 3.4 Validation of the efficacy and utility of dbGVOSCC

We compared the effectiveness of dbGVOSCC and ChatGPT-5 in retrieving relevant information from patient data within scientific and clinical research contexts (**Supplementary Material S1**). Both systems performed well in matching queries with relevant responses (9.63 vs. 10). However, compared with ChatGPT-5, dbGVOSCC demonstrated superior performance in several key aspects: providing detailed information from research articles (0.41 vs. 9.6), recommending publications with accessible links (0.26 vs. 1.3), achieving a higher authenticity ratio of scientific articles (48.83 vs. 100), identifying original research articles (0.93 vs. 1.3), and delivering standardized output (0 vs. 100) (**Table 3**, **Supplementary Table S2**).

**Table 3:** Comparison between ChatGPT-5 and dbGVOSCC on question response.

Question ID	Matching between questions and responses	Present the details of the research articles	Number of recommended publications	Number of recommended publications with links	Scientific article authenticity ratio (%)	Number of original research	Standardized output results
ChatGPT-5							
1	9.333	1	3.333	0.667	70	2	N
2	10	0	2.667	0.667	50	1	N
3	9.333	0.667	2	0	22.222	0.667	N
4	9.333	0	1	0	22.222	0.667	N
5	9.333	0.667	2	0	72.222	1.333	N
6	10	0.333	3	1	88.889	0.667	N
7	10	0	2.333	0	58.333	1.333	N
8	10	0.333	0.333	0	33.333	0	N
9	9.333	0.667	1	0	22.222	0.667	N
10	10	0	3	0	100	3	N
Mean	9.63 ± 0.33	0.41 ± 0.35	1.96 ± 0.95	0.26 ± 0.37	48.83 ± 27.23	0.93 ± 0.81	0
dbGVOSCC							
1	10	10	1	1	100	1	Y
2	10	10	1	1	100	1	Y
3	10	10	2	2	100	2	Y
4	10	8	1	1	100	1	Y
5	10	10	1	1	100	1	Y
6	10	10	2	2	100	2	Y
7	10	10	1	1	100	1	Y
8	10	10	1	1	100	1	Y
9	10	8	1	1	100	1	Y
10	10	10	2	2	100	2	Y
Mean	10	9.6 ± 0.83	1.3 ± 0.47	1.3 ± 0.47	100	1.3 ± 0.47	100

**Figure 6:**
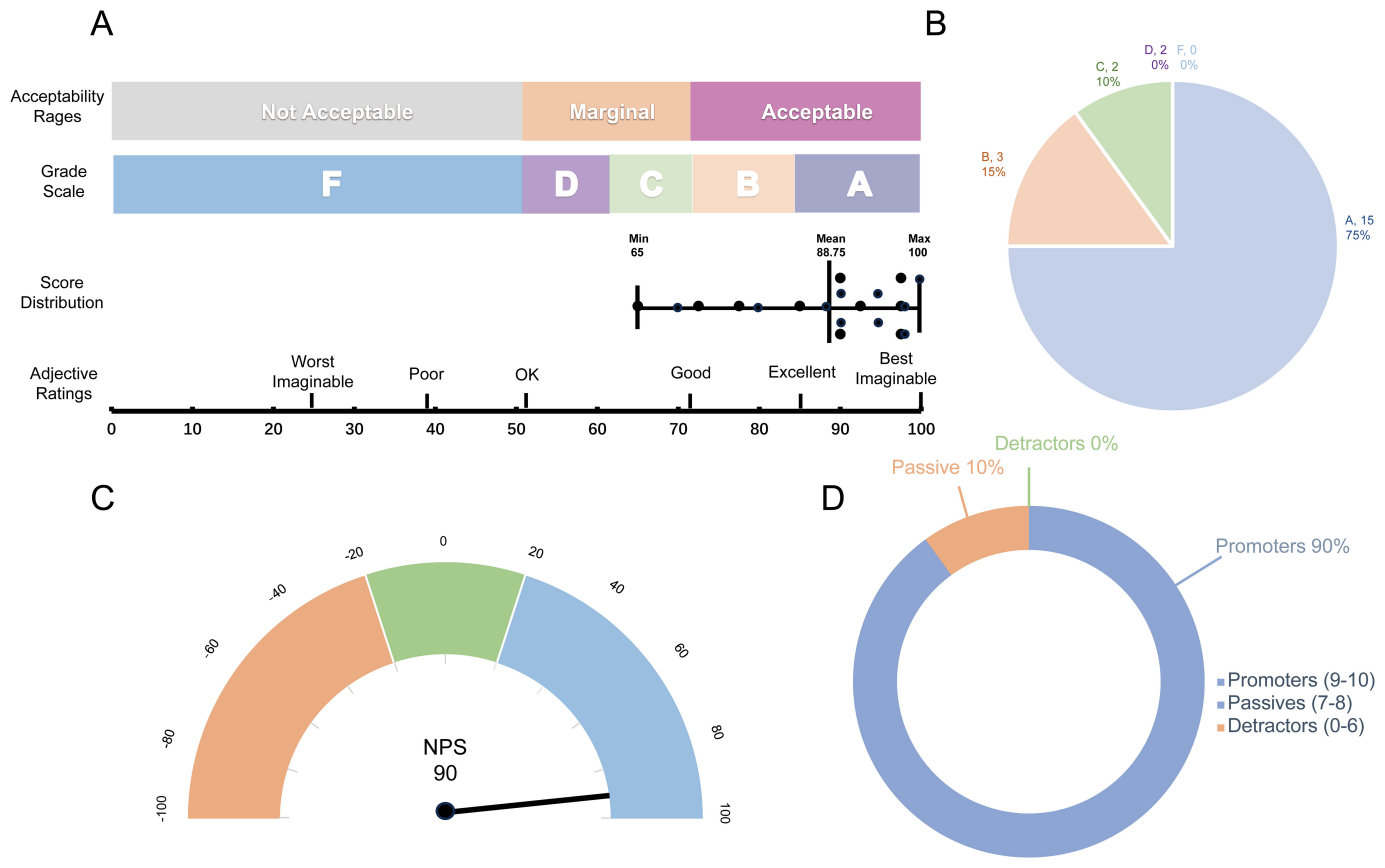
Validation results of dbGVOSCC. **(A)** Results of the System Usability Scale (SUS), including the grade scale, score distribution across 20 questionnaires, and corresponding adjective ratings. Each data point in the graph represents an individual user’s score, with the mean score of 88.75. **(B)** Grade distribution of the SUS, where Grade A and B account for 75% and 15% of users, respectively. **(C)** Net Promoter Score (NPS) results, showing an overall score of 90. **(D)** Distribution of scores on the NPS scale. According to the standard classification criteria, each user score falls into one of three categories; in this study, the percentage of detractors was zero.

dbGVOSCC achieved a score of 88.75 on the System Usability Scale (SUS), with adjective ratings classified as “Excellent”, a grade scale of “A”, and an acceptability range of “Acceptable”. These results indicate that dbGVOSCC demonstrates strong usability for users. Similarly, in the Net Promoter Score (NPS) assessment, dbGVOSCC scored 90, with 90% of participants identified as promoters, 10% as passive, and none as detractors. This further underscores the high level of user satisfaction with dbGVOSCC (**Figure 6**).

We also compared the dbGVOSCC with other databases relevant to oral OSCC, focusing on sample size, population diversity, disease specificity, and clinical annotations. As shown in **Table 4**, these comparisons underscore the distinctive scope and utility of dbGVOSCC as the most comprehensive, OSCC-specific genomic resource currently available.

**Table 4:** Comparison of OSCC-related databases.

Database	Year	Sample Size (OSCC-related)	Population diversity	OSCC-specific	Clinical annotation	Key notes
dbGVOSCC (This work)	2024	106,079	Multi-ethnic (Asia, Europe, Americas)	**√**	**√** Full: age, sex, site, geography	Comprehensive, population-diverse, clinically rich OSCC database
dbGENVOC	2021	100 WES + 5 WGS (India) + 220 TCGA + 118 literature = 443	Indian + US (TCGA) + global publications	**√** Partial	**×** Minimal	Mainly Indian WES/WGS + integrated TCGA/literature mutations
TCGA-HNSC	~2015	~315–391 OSCC subset (of 528 HNSC)	North American (mainly Caucasian)	**×**	**√** Survival, pathology, treatment	Subset of HNSC cohort with OSCC tumors, strong clinical annotation
cBioPortal (HNSC)	Ongoing	~528 (matches TCGA-HNSC)	Same as TCGA	**×**	**√**	Interactive platform accessing TCGA and other HNSC data
ICGC (OSCC projects)	~2020	57 OSCC samples	South Asian and South American	**×** Partial	**√** Limited	Includes data from Indian and Brazilian OSCC projects
OncoMX	2023	Not defined (meta-database)	Global (TCGA, ICGC, GTEx)	**×**	**√** Aggregated clinical/biomarker data	Meta-resource integrating multi-platform cancer genomics data

√, Feature present/available for that database.

## 4 Discussion

In this study, we established dbGVOSCC, a manually curated knowledge base that systematically integrates 1,788 entries of somatic genetic variations from 106,079 clinical samples of 400 peer-reviewed studies. By encompassing a wide spectrum of mutation types, including SNPs, point mutations, indels, CNVs, LOH, and epimutations, dbGVOSCC provides a more comprehensive and structured resource than existing OSCC-related databases. Notably, it demonstrates higher accuracy and more organized output results when extracting information from scientific literature compared with ChatGPT-5, while its high SUS (88.75) and NPS (90) further underscore its reliability and usability. Together, these attributes position dbGVOSCC as a robust platform for systems genetics research and a valuable tool for advancing precision oncology in OSCC.

The dbGVOSCC platform was developed to address the pressing need for accessible, structured, and high-quality data in OSCC research. With its seven functional modules, including advanced search tools, classification browsing, and regular updates, dbGVOSCC not only facilitates the exploration of OSCC-related genetic variations but also bridges the gap between raw genetic data and actionable clinical insights. As a first step toward addressing OSCC heterogeneity, dbGVOSCC already extends beyond SNVs to include CNVs and LOH, and it provides clinical and topographic metadata (tumor subsite, age, sex, sample source, country), enabling stratified analyses relevant to heterogeneity. This user-friendly, open-access platform empowers researchers and clinicians alike, enhancing the translational application of genetic information in diagnostics, therapeutics, and prognostics.

Bioinformatics analyses provided comprehensive insights into the biological processes, cellular components, and molecular functions underlying OSCC pathogenesis. GO enrichment revealed that OSCC-related genes play key roles in tumor growth, immune modulation, and signaling pathways, particularly cell proliferation, leukocyte differentiation, and the MAPK cascade (29). Cellular component enrichment highlighted receptor and adhesion-related protein complexes essential for signal transduction and tumor-microenvironment interactions. While molecular function analysis emphasized tyrosine kinase activity and p53 binding, pointing to potential therapeutic targets (30). KEGG pathway analysis further identified the PI3K-Akt pathway, general cancer pathways, and other malignancy-specific pathways (e.g., melanoma, bladder cancer, breast cancer) as central to OSCC progression (31). These shared mechanisms suggest opportunities for cross-cancer therapeutic strategies, with the PI3K/mTOR pathway already showing promise through inhibitors such as BKM120 and BYL719, which act as radiosensitizers in OSCC (32). Disease-gene and phenotype enrichment analyses underscored the broader impact of OSCC-related genetic variations, revealing overlap with thoracic, breast, and gastrointestinal cancers and associations with gonadal, ovarian, and skin neoplasms (33, 34). Tissue expression analysis further demonstrated links to cervical carcinoma cells, regulatory T lymphocytes, and leukemia cells (35), highlighting the dual roles of OSCC-related genes in tumor-specific and immune-related processes. Collectively, these findings emphasize the importance of targeting both tumor biology and the tumor microenvironment to enhance treatment efficacy and reduce resistance.

Despite the rapid advancement of large language models (LLMs) such as ChatGPT in biomedical knowledge extraction, their utility in clinical and translational research remains limited by fundamental challenges, including unverifiable data sources, generalized knowledge representations, and the hallucination phenomenon (36). LLMs are typically trained on heterogeneous, non-curated corpora, leading to compromised data traceability and an elevated risk of generating fabricated or inaccurate information. Just as in the comparison results of ChatGPT-5, our database exhibits superior accuracy and more structured output, further supported by high SUS and NPS. Such limitations are particularly problematic for applications in precision oncology, where the fidelity, granularity, and clinical validity of data are paramount.

In contrast, dbGVOSCC, our curated knowledge base focused on genetic variants in OSCC, was meticulously constructed through manual extraction from peer-reviewed scientific literature, ensuring the authenticity, accuracy, and traceability of every entry. Compared with existing OSCC-related databases such as dbGENVOC and OncoMX (37), dbGVOSCC incorporates a more comprehensive and continuously updated collection of data. Beyond basic variant annotation, dbGVOSCC systematically captures critical clinical metadata, including anatomical tumor subsite, patient age, sample source, sex, and geographic origin, enabling stratified analyses of genetic variation patterns across diverse populations. Recognizing that genetic predisposition and somatic mutation spectra vary substantially across ethnicities, regions, and lifestyle backgrounds, dbGVOSCC offers a level of contextual specificity not achievable by generalized LLM outputs.

Moreover, its strictly curated and continuously updated architecture circumvents the issue of model collapse that affects LLMs trained on self-generated data, ensuring dbGVOSCC remains a reliable and clinically actionable resource over time. Collectively, these attributes position dbGVOSCC as a more specialized, precise, and trustworthy platform for advancing systems genetics research and facilitating the development of targeted therapies for oral squamous cell carcinoma. Furthermore, dbGVOSCC may serve as a foundation for training specialized machine learning models, leveraging curated, high-fidelity datasets to overcome the hallucination and data contamination challenges associated with general-purpose LLMs (38). By maintaining a rigorous curation standard while embracing computational advances, dbGVOSCC is poised to support future efforts in precision oncology, including biomarker discovery, therapeutic target identification, and personalized treatment strategy development for oral squamous cell carcinoma (39).

### 4.1 Future directions and challenges

While dbGVOSCC provides a robust platform for exploring OSCC-related genetic variations, some limitations remain. Tumor heterogeneity, a hallmark of malignancies, is not yet fully captured in the database. Intratumoral heterogeneity and genomic instability, which contribute to tumor progression and therapeutic resistance, are areas that require further data integration. Addressing these gaps will be a priority in future updates.

**Figure 7:**
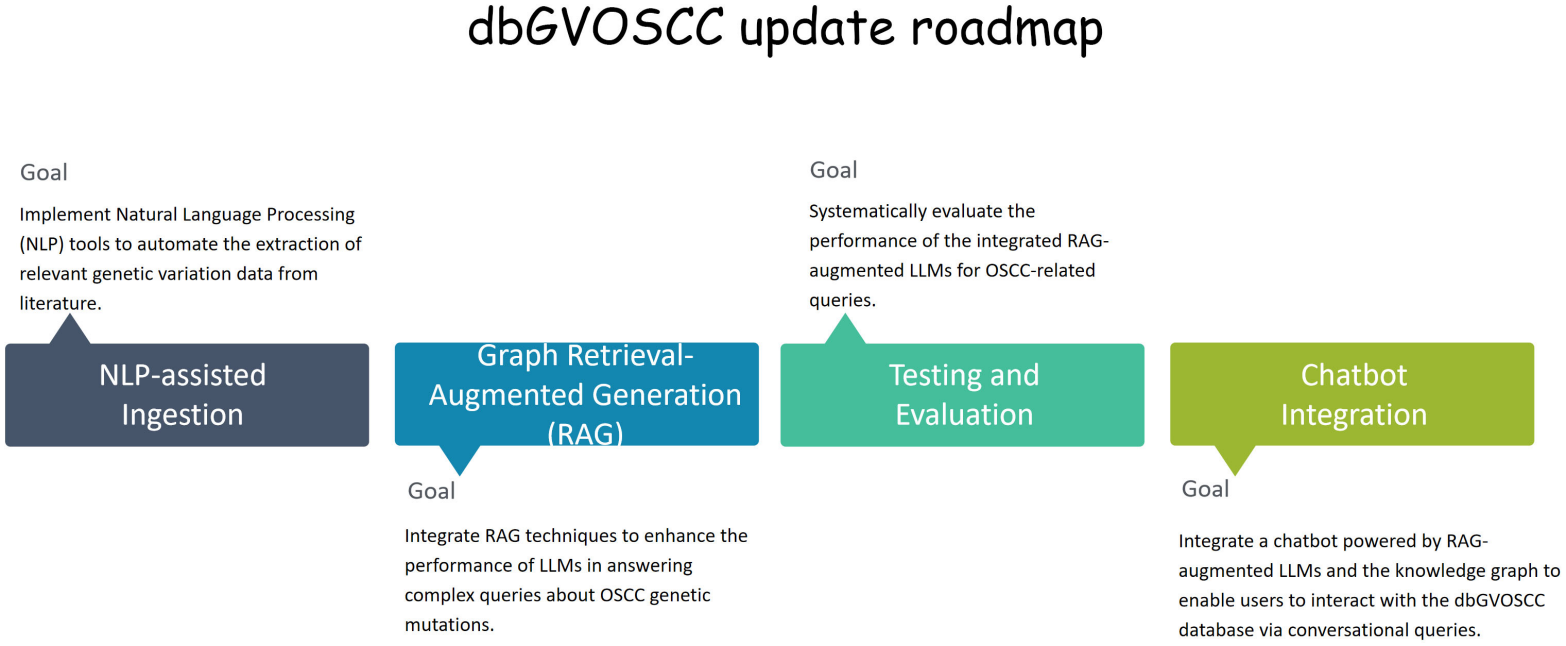
The dbGVOSCC update roadmap. This roadmap delineates the principal milestones and timelines for embedding NLP tools and knowledge graphs within the dbGVOSCC database. It encompasses key stages, including knowledge graph construction, NLP-facilitated data ingestion, seamless integration with RAG models to optimize query generation, and culminating in a chatbot deployment tailored for clinical and research support.

We plan to enhance the representation of OSCC complexities and heterogeneity in future updates of dbGVOSCC by: 1) incorporating finer-grained intertumoral architecture through NLP-assisted literature curation; 2) developing an OSCC-specific ontology to standardize heterogeneous features; 3) converting the relational database into a knowledge graph, allowing systematic capture and integration of genomic instability patterns (**Figure 7**). These advancements will enhance the platform’s usability and applicability in translational medicine. Moreover, the integration of a knowledge graph with medical chatbots could facilitate more effective doctor-patient communication, improving clinical decision-making and personalized care.

In conclusion, this study highlights the complex molecular mechanisms underlying OSCC and demonstrates the utility of dbGVOSCC as a resource for advancing research and clinical applications. By integrating genomic, clinical, and functional data, dbGVOSCC not only provides a comprehensive tool for understanding OSCC pathogenesis but also sets the stage for developing novel diagnostic tools, therapeutic strategies, and prognostic models. Continued updates and expansions of the database will ensure its relevance and utility in the rapidly evolving field of precision oncology.

## Data Availability

The original contributions presented in the study are included in the article/**Supplementary Material**. Further inquiries can be directed to the corresponding authors.

## Supplementary material

Supplementary Figure 1 Distribution of OSCC samples by primary oral topographic sites. **(A)** Illustrations of 7 different topographic locations in the oral cavity; **(B)** 13, 083 samples mainly distributed in 7 different oral topographic sites.

Supplementary Figure 2 The dbGVOSCC database variant gene enrichment analysis. **(A)** Biological Process; **(B)** Cellular Component; **(C)** Molecular Function; **(D)** KEGG pathways. Significance by hypergeometric test with Benjamini-Hochberg FDR; only FDR < 0.05 and fold enrichment > 2 terms are shown. Dot color = -log^10^(FDR), dot size = hit gene count, x-axis = gene ratio.
