# Supplementary material for: dbGVOSCC: a comprehensive database of genetic variation for systems genetics research on oral squamous cell carcinoma
Source: Front Oncol. 2025 Oct 31;15:1692732. doi: 10.3389/fonc.2025.1692732 (PMC12616376; doi:10.3389/fonc.2025.1692732)

**Supplementary S1 The original output results of ChatGPT and dbGVOSCC for the questions**

**ChatGPT-5 output**

I have some information about oral squamous cell carcinoma (OSCC) genetic variations. Based on the provided information, could you help me understand the association between these genetic variations and OSCC? Additionally, please share relevant scientific literature that includes information about these variations.

1. The information is: TP53P177L mutation is a likely pathogenic variant linked to oral squamous cell carcinoma (OSCC).
2. The information is: KRAS CNV mutation is a likely pathogenic variant linked to oral squamous cell carcinoma (OSCC).
3. The information is: rs1138272 mutation is a likely pathogenic variant linked to oral squamous cell carcinoma (OSCC).
4. The information is: RB1CC1 homozygous deletion mutation is a likely pathogenic variant linked to oral squamous cell carcinoma (OSCC).
5. The information is: rs6950683 mutation is a likely benign variant linked to oral squamous cell carcinoma (OSCC).
6. The information is: HIF-1a C1772T polymorphism mutation is a pathogenic variant linked to oral squamous cell carcinoma (OSCC).
7. The information is: NOTCH1 c.842 mutation is a pathogenic variant linked to oral squamous cell carcinoma (OSCC).
8. The information is: EGFR CA repeats polymorphism mutation is a pathogenic variant linked to oral squamous cell carcinoma (OSCC).
9. The information is: TGFBR2 receptor II mutation is a pathogenic variant linked to oral squamous cell carcinoma (OSCC).
10. The information is: NOTCH1 C1133Y mutation is a pathogenic variant linked to oral squamous cell carcinoma (OSCC).

1.1

I have some information about oral squamous cell carcinoma (OSCC) genetic variations. Based on the provided information, could you help me understand the association between these genetic variations and OSCC? Additionally, please share relevant scientific literature that includes information about these variations.

The information is: TP53P177L mutation is a likely pathogenic variant linked to oral squamous cell carcinoma (OSCC).

The TP53P177L mutation is a likely pathogenic variant associated with oral squamous cell carcinoma (OSCC). This mutation affects the TP53 gene, which encodes the p53 protein—a crucial tumor suppressor involved in regulating cell cycle progression, apoptosis, and DNA repair. Mutations in TP53 can impair its normal function, leading to uncontrolled cell proliferation and tumorigenesis.

**Association Between TP53P177L Mutation and OSCC**

The TP53P177L mutation has been classified as likely pathogenic based on several lines of evidence:

- **Functional Impact**: Functional studies have demonstrated that the TP53P177L variant impairs the transcriptional activity, DNA binding, and apoptosis functions of the p53 protein. These disruptions are consistent with the loss-of-function mechanism observed in many cancer-associated TP53 mutations. [国家生物技术信息中心](https://www.ncbi.nlm.nih.gov/clinvar/variation/VCV000141764?utm_source=chatgpt.com) YES
- **Clinical Observations**: This mutation has been reported in individuals with clinical features of Li-Fraumeni syndrome, a hereditary cancer predisposition syndrome, indicating its potential role in cancer development. [国家生物技术信息中心](https://www.ncbi.nlm.nih.gov/clinvar/variation/VCV000141764?utm_source=chatgpt.com)YES
- **Population Data**: The TP53P177L variant is not commonly observed in large population cohorts, suggesting it is a rare variant with potential pathogenic significance. [国家生物技术信息中心](https://www.ncbi.nlm.nih.gov/clinvar/variation/VCV000141764?utm_source=chatgpt.com)YES

In OSCC, TP53 mutations are prevalent and often correlate with poor prognosis. Specifically, high-risk TP53 mutations have been associated with increased chances of extranodal extension (ENE) and reduced overall and disease-free survival. The presence of such mutations may influence treatment decisions and patient management strategies. [PubMed+2AACR Journals+2](https://pubmed.ncbi.nlm.nih.gov/29330202/?utm_source=chatgpt.com) YES

**Relevant Scientific Literature**

Here are some key studies that discuss the role of TP53 mutations, including TP53P177L, in OSCC:

1. **High-Risk TP53 Mutations Are Associated with Extranodal Extension in Oral Cavity Squamous Cell Carcinoma**
   This study identifies a subgroup of TP53 mutations associated with increased risk of ENE in OSCC patients, highlighting the clinical relevance of TP53 mutation status. YES 18
2. **Functionally Impactful TP53 Mutations Are Associated with Increased Risk of Extranodal Extension in Patients with Advanced OSCC**
   This research emphasizes the association between high-risk TP53 mutations and ENE, suggesting that TP53 mutation status may serve as a potential biomarker for treatment decisions. YES 20
3. **TP53 Gain-of-Function Mutation Modulates the Immunosuppressive Tumor Microenvironment in OSCC**
   This study explores how specific TP53 mutations can alter the tumor immune microenvironment, potentially impacting tumor progression and response to therapy. Feak
4. **The Mutational Spectrum in Whole Exon of p53 in Oral Squamous Cell Carcinoma**
   This comprehensive analysis of TP53 mutations in OSCC provides insights into the diversity of mutations and their potential functional consequences. Feak
5. **Specific p53 Mutations Predict Poor Prognosis in Oral Squamous Cell Carcinoma**
   This study focuses on the correlation between specific p53 mutations and clinical outcomes in OSCC patients, underscoring the prognostic value of TP53 mutation analysis. YES 03

**Conclusion**

The TP53P177L mutation is a likely pathogenic variant that contributes to the development and progression of OSCC. Its impact on p53 function underscores the importance of TP53 as a tumor suppressor gene in oral carcinogenesis. Understanding the role of specific TP53 mutations, including TP53P177L, can aid in prognostication and may inform therapeutic strategies for OSCC patients.

1.2

I have some information about oral squamous cell carcinoma (OSCC) genetic variations. Based on the provided information, could you help me understand the association between these genetic variations and OSCC? Additionally, please share relevant scientific literature that includes information about these variations.

The information is: TP53P177L mutation is a likely pathogenic variant linked to oral squamous cell carcinoma (OSCC).

The TP53P177L mutation is a variant in the TP53 gene, which encodes the tumor suppressor protein p53. This protein plays a crucial role in regulating the cell cycle, DNA repair, and apoptosis. Mutations in TP53 are commonly associated with various cancers, including oral squamous cell carcinoma (OSCC).

### Association Between TP53P177L Mutation and OSCC

While specific studies on the TP53P177L mutation in OSCC are limited, TP53 mutations in general are prevalent in head and neck squamous cell carcinoma (HNSCC), which includes OSCC. These mutations often lead to a loss of normal p53 function, contributing to tumorigenesis.

A study by Lindemann et al. (2018) discussed how TP53 mutations are associated with poor survival and resistance to radiotherapy and chemotherapy in OSCC patients. This highlights the potential impact of TP53 mutations on treatment outcomes in OSCC. ([PubMed][1])

Additionally, research by Gleber-Netto et al. (2020) found that high-risk TP53 mutations are linked to an increased chance of detecting extranodal extension (ENE) in patients with advanced OSCC. ENE is a significant factor in determining the prognosis and treatment strategies for OSCC patients. ([PubMed][2])

### Clinical Implications

The presence of a likely pathogenic TP53 mutation, such as TP53P177L, may have several clinical implications:

* **Prognostic Indicator**: Mutations in TP53 can serve as biomarkers for poor prognosis, indicating a higher likelihood of tumor progression and metastasis.

* **Therapeutic Resistance**: OSCC tumors harboring TP53 mutations may exhibit resistance to standard treatments, including chemotherapy and radiotherapy.

* **Personalized Treatment Strategies**: Identifying specific TP53 mutations can aid in developing personalized treatment plans, potentially incorporating targeted therapies that address the specific mutation.

### Further Reading

For more detailed information on the role of TP53 mutations in OSCC, consider reviewing the following studies:

* Lindemann et al. (2018): This review discusses the association between TP53 mutations and treatment outcomes in OSCC patients. ([PubMed][1])

* Gleber-Netto et al. (2020): This study explores the link between high-risk TP53 mutations and extranodal extension in OSCC. ([PubMed][2])

These resources provide insights into the molecular mechanisms by which TP53 mutations influence OSCC pathogenesis and treatment response.

If you have access to specific patient data or additional genetic information, further analysis could be conducted to assess the impact of the TP53P177L mutation on OSCC progression and treatment outcomes.

[1]: https://pubmed.ncbi.nlm.nih.gov/29489434/?utm_source=chatgpt.com "Targeting the DNA Damage Response in OSCC with TP53 ..." Yes, review, 18

[2]: https://pubmed.ncbi.nlm.nih.gov/32797678/?utm_source=chatgpt.com "Functionally impactful TP53 mutations are associated with ..." Yes，article，20

1.3

I have some information about oral squamous cell carcinoma (OSCC) genetic variations. Based on the provided information, could you help me understand the association between these genetic variations and OSCC? Additionally, please share relevant scientific literature that includes information about these variations.

The information is: TP53P177L mutation is a likely pathogenic variant linked to oral squamous cell carcinoma (OSCC).

The TP53P177L mutation is a likely pathogenic variant associated with oral squamous cell carcinoma (OSCC). This mutation affects the TP53 gene, which encodes the p53 protein—a crucial tumor suppressor involved in regulating the cell cycle, DNA repair, and apoptosis. Mutations in TP53 can impair these functions, leading to uncontrolled cell proliferation and tumorigenesis.

Association Between TP53 Mutations and OSCC

TP53 mutations are among the most common genetic alterations in OSCC. These mutations are often linked to poor prognosis and resistance to treatments like chemotherapy and radiotherapy. High-risk TP53 mutations, such as those affecting the DNA-binding domain, are particularly associated with aggressive disease characteristics, including extranodal extension (ENE), which is a negative prognostic factor in OSCC .

The Evolutionary Action score of p53 (EAp53) is a computational tool that classifies TP53 mutations based on their potential to disrupt p53 function. Mutations with high EAp53 scores are considered high-risk and are associated with increased chances of detecting ENE in OSCC patients .

Clinical Implications

The presence of high-risk TP53 mutations can influence treatment decisions in OSCC. For instance, patients with tumors harboring such mutations may benefit from more aggressive treatment strategies, including postoperative adjuvant therapy, to address the increased risk of ENE and potential treatment resistance .

For a more in-depth understanding, consider reviewing the following studies:

Sandulache VC, et al. (2018): This study discusses the association between high-risk TP53 mutations and ENE in OSCC, highlighting the potential of TP53 mutation status as a prognostic biomarker . yes,article

Gleber-Netto FO, et al. (2020): This research investigates the impact of functionally impactful TP53 mutations on ENE in OSCC, providing insights into the role of TP53 in tumor progression . yes,article

PubMed

Lindemann A, et al. (2018): This review examines the role of TP53 mutations in the DNA damage response in OSCC, discussing potential therapeutic strategies targeting these mutations . Yes review,

PubMed

These studies underscore the significance of TP53 mutations in OSCC and their potential utility in guiding clinical management.

2.1

I have some information about oral squamous cell carcinoma (OSCC) genetic variations. Based on the provided information, could you help me understand the association between these genetic variations and OSCC? Additionally, please share relevant scientific literature that includes information about these variations.

The information is: KRAS CNV mutation is a likely pathogenic variant linked to oral squamous cell carcinoma (OSCC).

KRAS copy number variation (CNV) mutations have been identified as likely pathogenic variants associated with oral squamous cell carcinoma (OSCC). These alterations contribute to the dysregulation of critical signaling pathways, thereby promoting tumorigenesis and influencing clinical outcomes.

**Association Between KRAS CNV Mutations and OSCC**

KRAS is a pivotal oncogene involved in the regulation of the mitogen-activated protein kinase (MAPK) and phosphatidylinositol 3-kinase (PI3K) pathways, which are crucial for cell proliferation, survival, and differentiation. Amplifications or deletions of the KRAS gene can lead to its overexpression or loss of function, respectively, disrupting normal cellular processes and contributing to cancer development

In OSCC, studies have reported that KRAS amplifications are associated with shorter overall survival, indicating their potential role in tumor progression and poor prognosis. Additionally, the presence of KRAS mutations has been linked to increased resistance to certain therapies, underscoring the need for targeted treatment strategies.

**Scientific Literature on KRAS CNV Mutations in OSCC**

1. **Molecular Profiles of Response to Neoadjuvant Chemotherapy in OSCC**
   This study found that KRAS amplification (15%) was associated with shorter overall survival in OSCC patients, suggesting its potential as a prognostic biomarker. Feak
2. **Mechanisms of Resistance to Oncogenic KRAS Inhibition**
   This review discusses how KRAS mutations, including amplifications, can lead to resistance against targeted therapies, highlighting the complexity of treating cancers harboring KRAS alterations. feak
3. **Copy Number Variation in OSCC**
   This study identified recurrent copy number alterations in OSCC, including amplifications of genes like KRAS, which may contribute to tumorigenesis and could serve as therapeutic targets. feak

**Conclusion**

KRAS CNV mutations play a significant role in the pathogenesis of OSCC by disrupting key signaling pathways that regulate cell growth and survival. Their presence is associated with poor prognosis and resistance to certain therapies, highlighting the importance of KRAS as a potential biomarker and therapeutic target in OSCC. Further research is needed to develop targeted therapies that can effectively address KRAS-driven malignancies.

2.2

I have some information about oral squamous cell carcinoma (OSCC) genetic variations. Based on the provided information, could you help me understand the association between these genetic variations and OSCC? Additionally, please share relevant scientific literature that includes information about these variations.

The information is: KRAS CNV mutation is a likely pathogenic variant linked to oral squamous cell carcinoma (OSCC).

The KRAS gene encodes a GTPase protein that plays a pivotal role in cell signaling pathways, including those regulating cell growth, differentiation, and survival. Mutations in KRAS, particularly those leading to its constitutive activation, are well-documented in various cancers. In the context of oral squamous cell carcinoma (OSCC), while somatic mutations in KRAS are less frequently observed compared to other genes like TP53, the presence of KRAS copy number variations (CNVs) has been noted.

KRAS CNV and OSCC

A study by Koo et al. (2021) identified DNA copy-number aberrations in 64% of OSCC tumors, with amplifications in genes such as EGFR, MMP12, and PRKDC. However, KRAS was not among the most frequently altered genes in this cohort. This suggests that while KRAS alterations may occur in OSCC, they are not as prevalent as alterations in other genes. (cannot be determined)

MDPI

Another study by Huang et al. (2024) reviewed 20 studies comparing mutational profiles between tumor tissue DNA and circulating tumor DNA in head and neck squamous cell carcinoma (HNSCC) patients, which includes OSCC. (Yes,article) While this review provides insights into the mutational landscape of HNSCC, specific data on KRAS CNVs in OSCC were not detailed.

Clinical Implications

The presence of KRAS CNVs in OSCC could have potential clinical implications. KRAS mutations are known to affect response to certain therapies in other cancers. For instance, in colorectal cancer, KRAS mutations can predict resistance to EGFR-targeted therapies. While direct evidence linking KRAS CNVs to treatment outcomes in OSCC is limited, understanding the role of KRAS alterations could inform therapeutic strategies and prognostic assessments.

Conclusion

In summary, while KRAS CNVs are less frequently observed in OSCC compared to other genetic alterations, their potential role in the pathogenesis and clinical management of OSCC warrants further investigation. Continued research into the genetic landscape of OSCC will enhance our understanding and may lead to more targeted and effective treatment approaches.

2.3

I have some information about oral squamous cell carcinoma (OSCC) genetic variations. Based on the provided information, could you help me understand the association between these genetic variations and OSCC? Additionally, please share relevant scientific literature that includes information about these variations.

The information is: KRAS CNV mutation is a likely pathogenic variant linked to oral squamous cell carcinoma (OSCC).

The KRAS gene is a pivotal proto-oncogene involved in regulating cell proliferation and survival through the RAS/MAPK signaling pathway. Mutations or copy number variations (CNVs) in KRAS can lead to its constitutive activation, contributing to oncogenesis in various cancers, including oral squamous cell carcinoma (OSCC).

**KRAS CNV and Its Role in OSCC**

Copy number variations in KRAS, such as amplifications or deletions, can influence the gene's expression levels. In the context of OSCC, these alterations may disrupt normal cellular signaling, promoting tumorigenesis. While direct studies linking KRAS CNV to OSCC are limited, research in other cancers suggests that KRAS amplifications can impact tumor behavior and treatment responses.

**Clinical Implications**

- **Prognostic Significance**: In non-small cell lung cancer (NSCLC), the presence of KRAS mutations combined with CNV has been associated with poorer survival outcomes. Specifically, KRAS mutant tumors with copy number gain exhibited worse overall survival compared to those without such alterations.
- **Predictive Value**: Interestingly, in the adenocarcinoma subtype of NSCLC, patients with KRAS mutations and CNV showed a more favorable response to chemotherapy, indicating that KRAS CNV might influence treatment efficacy.

**Scientific Literature on KRAS Variants in OSCC**

While specific studies on KRAS CNV in OSCC are scarce, several articles discuss the broader implications of KRAS mutations and alterations in head and neck cancers:

- **Somatic Mutation Detection and KRAS Amplification in OSCC**: This study emphasizes the importance of detecting somatic mutations and amplifications in KRAS for understanding their role in OSCC pathogenesis.
- **Profiling Cancer-Related Gene Mutations in OSCC**: Comprehensive genomic analyses highlighted the significance of various mutations, including those in KRAS, in the development of OSCC.

**Conclusion**

KRAS CNV mutations are likely pathogenic variants associated with OSCC, influencing tumor behavior and potentially affecting treatment responses. Further research is needed to elucidate the precise mechanisms by which KRAS CNVs contribute to OSCC and to explore their potential as therapeutic targets.

For a more in-depth understanding, the following articles provide valuable insights:

- [Somatic Mutation Detection and KRAS Amplification in OSCC](https://pmc.ncbi.nlm.nih.gov/articles/PMC10060882/)[PMC+3PMC+3PMC+3](https://pmc.ncbi.nlm.nih.gov/articles/PMC10060882/?utm_source=chatgpt.com),23
- [Profiling Cancer-Related Gene Mutations in OSCC](https://pmc.ncbi.nlm.nih.gov/articles/PMC5601718/)[PMC](https://pmc.ncbi.nlm.nih.gov/articles/PMC5601718/?utm_source=chatgpt.com),17
- [Prognostic and Predictive Effect of KRAS Gene Copy Number in NSCLC](https://pmc.ncbi.nlm.nih.gov/articles/PMC7947394/)[PMC+1](https://pmc.ncbi.nlm.nih.gov/articles/PMC7947394/?utm_source=chatgpt.com),21

If you require assistance in accessing these articles or further information on KRAS-related research in OSCC, feel free to ask.

3.1

I have some information about oral squamous cell carcinoma (OSCC) genetic variations. Based on the provided information, could you help me understand the association between these genetic variations and OSCC? Additionally, please share relevant scientific literature that includes information about these variations.

The information is: rs1138272 mutation is a likely pathogenic variant linked to oral squamous cell carcinoma (OSCC).

The rs1138272 polymorphism, a single nucleotide variation (C341T) in the GSTP1 gene, has been implicated in various cancers, including oral squamous cell carcinoma (OSCC). This variant leads to an alanine (Ala) to valine (Val) substitution at codon 114, potentially affecting the enzyme's function.

Association Between rs1138272 and OSCC

While direct studies linking rs1138272 to OSCC are limited, its role in cancer susceptibility has been explored in other malignancies. A comprehensive meta-analysis encompassing 43 studies with over 15,000 cancer cases indicated that the TT genotype of rs1138272 is associated with increased cancer risk in Asian and African populations, and specifically with head and neck cancers in Caucasians .

The GSTP1 enzyme, influenced by rs1138272, is involved in the detoxification of carcinogens, including those found in tobacco smoke. Reduced enzymatic activity due to the Val114 variant may impair the detoxification process, thereby increasing susceptibility to cancers associated with tobacco exposure, such as OSCC.

Scientific Literature on rs1138272

Comprehensive Analysis of the Association Between rs1138272 and Cancer Risk, Feak

This meta-analysis found a significant association between the TT genotype of rs1138272 and increased cancer risk in Asian and African populations, and specifically with head and neck cancers in Caucasians .

GSTP1 rs1138272 Polymorphism and Prostate Cancer Risk Feak

A study reported that carriers of the Val114 variant allele (rs1138272) had a 4.93 times higher risk of prostate cancer compared to individuals with the reference alleles. This suggests that the variant may influence cancer susceptibility through gene-gene interactions .

GSTP1 rs1138272 Polymorphism Affects Prostate Cancer Risk Yes, article,20

Another study found that the GSTP1*Val (rs1138272) variant allele was associated with prostate cancer risk, highlighting the role of this polymorphism in cancer susceptibility .

Association Analysis of rs1695 and rs1138272 Variations in GSTP1 Gene and Breast Cancer Susceptibility

This research examined the association of rs1138272 with breast cancer risk, noting that while rs1695 was significantly associated with increased risk, rs1138272 did not show a significant association in the studied population .

Conclusion

The rs1138272 polymorphism in the GSTP1 gene appears to influence cancer susceptibility, including OSCC, by altering the enzyme's detoxification capacity. While direct studies on OSCC are limited, the association of rs1138272 with other cancers suggests its potential role in OSCC pathogenesis. Further research is needed to elucidate the specific impact of rs1138272 on OSCC risk and its potential as a biomarker for early detection and personalized treatment strategies.

3.2

I have some information about oral squamous cell carcinoma (OSCC) genetic variations. Based on the provided information, could you help me understand the association between these genetic variations and OSCC? Additionally, please share relevant scientific literature that includes information about these variations.

The information is: rs1138272 mutation is a likely pathogenic variant linked to oral squamous cell carcinoma (OSCC).

The rs1138272 polymorphism, also known as c.341C>T or Ala114Val, is a genetic variation in the GSTP1 gene, which encodes the glutathione S-transferase P1 enzyme. This enzyme plays a crucial role in detoxifying reactive oxygen species and electrophilic compounds, thereby protecting cells from oxidative damage. The rs1138272 variant involves a substitution of alanine (Ala) with valine (Val) at codon 114, potentially altering the enzyme's substrate specificity and enzymatic activity.

Association with Oral Squamous Cell Carcinoma (OSCC)

Research on the direct association between rs1138272 and OSCC is limited. However, studies have explored the role of GSTP1 polymorphisms, including rs1138272, in cancer susceptibility. For instance, a meta-analysis indicated that the TT genotype of the GSTP1 rs1138272 polymorphism is likely related to an increased susceptibility to overall cancer in Asian and African populations, and specifically to colorectal and head and neck cancers in Caucasian populations .

While this meta-analysis did not focus exclusively on OSCC, the inclusion of head and neck cancers suggests a potential relevance to OSCC. The altered enzymatic activity due to the rs1138272 variant may influence the detoxification processes in oral tissues, potentially affecting cancer risk.

Functional Implications

The GSTP1 rs1138272 polymorphism leads to an amino acid substitution at codon 114, which may affect the enzyme's ability to distinguish between planar and non-planar substrates. This change could influence the detoxification of various carcinogens, including those found in tobacco smoke, a known risk factor for OSCC .

For a comprehensive understanding of the association between rs1138272 and cancer susceptibility, including OSCC, consider reviewing the following studies:

Comprehensive Analysis of the Association Between GSTP1 rs1138272 Polymorphism and Cancer Susceptibility: This meta-analysis examines the relationship between the rs1138272 polymorphism and cancer risk across different populations . Yes,article,19 but one focus on OSCC

Association Analysis of rs1695 and rs1138272 Variations in GSTP1 Gene and Breast Cancer Susceptibility: Although focused on breast cancer, this study provides insights into the functional implications of the rs1138272 polymorphism in cancer susceptibility .Yes,article,20 but one focus on OSCC

GSTP1 rs1138272 Polymorphism Affects Prostate Cancer Risk: This research investigates the impact of the rs1138272 polymorphism on prostate cancer risk, offering comparative insights .

While these studies do not directly address OSCC, they highlight the potential role of the GSTP1 rs1138272 polymorphism in cancer susceptibility, warranting further research into its specific association with OSCC.Yes, article,20

3.3

I have some information about oral squamous cell carcinoma (OSCC) genetic variations. Based on the provided information, could you help me understand the association between these genetic variations and OSCC? Additionally, please share relevant scientific literature that includes information about these variations.

The information is: rs1138272 mutation is a likely pathogenic variant linked to oral squamous cell carcinoma (OSCC).

he rs1138272 single nucleotide polymorphism (SNP), located in the GSTP1 gene, results in an Ala114Val amino acid substitution. This variation has been implicated in various cancers, including oral squamous cell carcinoma (OSCC), due to its potential impact on the enzyme's detoxifying activity.

Association with OSCC

While rs1138272 has been studied in several cancers, specific studies focusing on its role in OSCC are limited. However, its involvement in other cancers suggests a possible relevance to OSCC. For instance, a meta-analysis indicated that the TT genotype of rs1138272 is associated with increased cancer susceptibility in Asian and African populations, and specifically with head and neck cancers in Caucasians.

Functional Implications

The Ala114Val substitution may alter the GSTP1 enzyme's ability to detoxify carcinogenic compounds, potentially leading to increased cancer risk. This functional change underscores the importance of studying rs1138272 in the context of OSCC.

Meta-analysis on GSTP1 rs1138272: This study found that the TT genotype of rs1138272 is associated with increased cancer susceptibility in Asian and African populations, and specifically with head and neck cancers in Caucasians.

GSTP1 rs1138272 and Prostate Cancer: Research indicates that the rs1138272 variant is associated with prostate cancer risk, highlighting its potential role in carcinogenesis.

Functional Analysis of GSTP1 Variants: Studies suggest that the rs1138272 variant may affect the GSTP1 enzyme's substrate specificity, potentially influencing cancer susceptibility.

Semantic Scholar

Conclusion

The rs1138272 SNP in the GSTP1 gene is a likely pathogenic variant that may influence OSCC risk through its effect on detoxification processes. Further research is needed to confirm its role in OSCC and to explore its potential as a biomarker for cancer susceptibility.

4.1

I have some information about oral squamous cell carcinoma (OSCC) genetic variations. Based on the provided information, could you help me understand the association between these genetic variations and OSCC? Additionally, please share relevant scientific literature that includes information about these variations.

The information is: RB1CC1 homozygous deletion mutation is a likely pathogenic variant linked to oral squamous cell carcinoma (OSCC).

The RB1CC1 gene encodes the RB1-inducible coiled-coil 1 protein, a key regulator of autophagy that interacts with the ULK1 complex to initiate autophagosome formation. This protein also influences cell cycle progression through interactions with the retinoblastoma protein (pRB) .

**Association Between RB1CC1 and OSCC**

Recent studies have highlighted the significance of RB1CC1 in oral squamous cell carcinoma (OSCC):

- **Autophagy Regulation**: RB1CC1 is involved in autophagy regulation, a process that can impact cancer cell survival and progression.
- **Expression Patterns**: In both human and mouse models of OSCC, RB1CC1 expression increases during the progression from normal mucosa to OSCC and lymph node metastases. Notably, its expression positively correlates with that of p53, a tumor suppressor protein.
- **Genetic Alterations**: Homozygous deletions of RB1CC1 have been observed in OSCC cell lines, suggesting its potential role as a tumor suppressor gene.

**Scientific Literature on RB1CC1 and OSCC**

Here are some key studies that explore the role of RB1CC1 in OSCC:

- **METTL14 Inhibits Malignant Progression of OSCC by Targeting RB1CC1**: This study demonstrates that METTL14, an m6A methyltransferase, regulates RB1CC1 expression through m6A modification, thereby influencing autophagy and progression in OSCC. Yes, article,23
- **Comparison of the Expression of TGF‑β1, E‑cadherin, N‑cadherin, TP53, RB1CC1, and HIF‑1α in OSCC and Lymph Node Metastases**: This research compares the expression levels of various biomarkers, including RB1CC1, across different stages of OSCC in humans and mice, highlighting its role in cancer progression. Yes, article,18
- **Early Discovery of Disseminated Tumor Cells During OSCC Progression**: The study identifies different levels of homozygous exon deletions in RB1CC1 and TP53 genes in disseminated tumor cells, suggesting their involvement in early tumor spread. Feak

These studies underscore the importance of RB1CC1 in OSCC pathogenesis and its potential as a biomarker for early detection and prognosis.

4.2

I have some information about oral squamous cell carcinoma (OSCC) genetic variations. Based on the provided information, could you help me understand the association between these genetic variations and OSCC? Additionally, please share relevant scientific literature that includes information about these variations.

The information is: RB1CC1 homozygous deletion mutation is a likely pathogenic variant linked to oral squamous cell carcinoma (OSCC).

The RB1CC1 homozygous deletion mutation is a likely pathogenic variant associated with oral squamous cell carcinoma (OSCC). This alteration disrupts the RB1CC1 gene, which encodes a protein involved in autophagy regulation and cell cycle control. Loss of RB1CC1 function can lead to uncontrolled cell proliferation and impaired cellular maintenance, contributing to OSCC development.

**Scientific Literature on RB1CC1 in OSCC:**

1. **Autophagy Regulation and Tumor Suppression:**
   RB1CC1 is essential for autophagosome formation, a process crucial for cellular homeostasis. In OSCC, METTL14, an m6A methyltransferase, regulates RB1CC1 expression, suggesting that RB1CC1-mediated autophagy suppression may inhibit OSCC progression . Feak
2. **Expression Patterns in OSCC:**
   Increased expression of RB1CC1 has been observed in various stages of OSCC in both human and mouse models. This upregulation correlates with the progression from normal mucosa to OSCC and lymph node metastases, indicating its potential role in tumor progression . Feak
3. **Genetic Deletions and Tumorigenesis:**
   Homozygous deletions involving RB1CC1 have been identified in OSCC cell lines, highlighting its potential as a tumor suppressor gene. The loss of RB1CC1 expression in these cell lines suggests that its deletion may contribute to oral carcinogenesis . Feak

**Conclusion:**

The RB1CC1 homozygous deletion mutation likely contributes to OSCC pathogenesis by impairing autophagy and cell cycle regulation. Further research is needed to fully elucidate its role and potential as a therapeutic target in OSCC.

4.3

I have some information about oral squamous cell carcinoma (OSCC) genetic variations. Based on the provided information, could you help me understand the association between these genetic variations and OSCC? Additionally, please share relevant scientific literature that includes information about these variations.

The information is: RB1CC1 homozygous deletion mutation is a likely pathogenic variant linked to oral squamous cell carcinoma (OSCC).

The RB1CC1 gene encodes a protein that plays a crucial role in autophagy, a cellular process that degrades and recycles components. Recent studies have highlighted the significance of RB1CC1 in oral squamous cell carcinoma (OSCC).

Key Findings:

Autophagy Regulation: RB1CC1 is essential for autophagosome formation. Its interaction with proteins like FIP200 and ULK1 is vital for initiating autophagy. Disruption in RB1CC1 function can impair autophagy, potentially leading to tumorigenesis.

Expression Patterns: In OSCC, RB1CC1 expression increases with cancer progression, suggesting its involvement in tumor development and metastasis. This upregulation correlates with the expression of p53, a tumor suppressor protein, indicating a potential cooperative role in cancer suppression.

Genetic Alterations: Homozygous deletions and mutations in RB1CC1 have been observed in OSCC, underscoring its potential as a tumor suppressor gene. Loss of RB1CC1 function may contribute to cancer progression by disrupting autophagic processes.

Scientific Literature:

Role of RB1CC1 in OSCC Progression: A study published in Oncology Letters examined the expression of RB1CC1 at different stages of OSCC in humans and mice. The results indicated that the expression of RB1CC1 increased in various stages of OSCC, suggesting its involvement in tumor progression. Furthermore, the expression of p53 was positively correlated with that of RB1CC1, highlighting a potential cooperative role in cancer suppression. Feak

Genetic Alterations in RB1CC1: Research has identified homozygous deletions and mutations in RB1CC1 in OSCC, implicating it as a potential tumor suppressor gene. These genetic alterations may lead to impaired autophagic processes, contributing to cancer progression. **Feak**

Conclusion:

RB1CC1 plays a significant role in the progression of OSCC through its involvement in autophagy regulation. Alterations in its expression and genetic makeup can disrupt normal cellular processes, contributing to tumorigenesis. Further research into RB1CC1's mechanisms may provide insights into potential therapeutic targets for OSCC.

5.1

I have some information about oral squamous cell carcinoma (OSCC) genetic variations. Based on the provided information, could you help me understand the association between these genetic variations and OSCC? Additionally, please share relevant scientific literature that includes information about these variations.

The information is: rs6950683 mutation is a likely benign variant linked to oral squamous cell carcinoma (OSCC).

The rs6950683 single nucleotide polymorphism (SNP) in the EZH2 gene has been studied for its potential role in oral squamous cell carcinoma (OSCC). Research indicates that the CC genotype of rs6950683 is associated with a reduced risk of developing OSCC compared to the wild-type TT genotype. This protective effect is thought to be due to hypermethylation of the EZH2 promoter region in individuals with the CC genotype, leading to decreased EZH2 expression .

EZH2 is a component of the Polycomb Repressive Complex 2 (PRC2), which is involved in gene silencing through histone methylation. Overexpression of EZH2 has been implicated in various cancers, including OSCC, by promoting cell proliferation and inhibiting differentiation. Therefore, reduced EZH2 expression resulting from rs6950683 CC genotype-associated hypermethylation may contribute to a lower risk of OSCC development .

In summary, the rs6950683 CC genotype is considered a likely benign variant in the context of OSCC, potentially offering a protective effect through epigenetic modulation of EZH2 expression.

For further reading, you may refer to the following scientific literature:

Su et al., "Effects of EZH2 promoter polymorphisms and methylation status on oral squamous cell carcinoma susceptibility and pathology," Oncotarget, 2016. Yes,article,16

Liu et al., "Analysis of EZH2 Genetic Variants on Triple-Negative Breast Cancer Susceptibility and Pathology," Int. J. Med. Sci., 2022. Yes, article,22

Chen et al., "Single nucleotide polymorphisms and cancer susceptibility," SciSpace, 2021. Feak(wrong author,journal,year)

These studies provide insights into the role of EZH2 genetic variants in cancer susceptibility and pathology.

5.2

I have some information about oral squamous cell carcinoma (OSCC) genetic variations. Based on the provided information, could you help me understand the association between these genetic variations and OSCC? Additionally, please share relevant scientific literature that includes information about these variations.

The information is: rs6950683 mutation is a likely benign variant linked to oral squamous cell carcinoma (OSCC).

The rs6950683 single-nucleotide polymorphism (SNP) in the EZH2 gene has been studied for its potential association with oral squamous cell carcinoma (OSCC). EZH2 is a histone methyltransferase that plays a crucial role in gene silencing and chromatin remodeling, processes that are often disrupted in cancer development.

**Association Between rs6950683 and OSCC**

Research indicates that the rs6950683 SNP may influence OSCC susceptibility. A study involving 576 OSCC patients and 552 healthy controls found that individuals carrying the CC genotype at rs6950683 had a lower risk of developing OSCC compared to those with the wild-type TT genotype. This association was further supported by the observation that the CC genotype exhibited higher methylation levels in the EZH2 promoter region, suggesting a potential mechanism for reduced gene expression and, consequently, a lower cancer risk .

**Functional Implications**

The CC genotype at rs6950683 is associated with increased methylation of the EZH2 promoter region. Hypermethylation typically leads to gene silencing, which in this context may reduce the expression of EZH2. Lower EZH2 expression could diminish its role in promoting tumorigenesis, thereby potentially decreasing the risk of OSCC development .

**Scientific Literature**

For a comprehensive understanding of the role of rs6950683 in OSCC, consider reviewing the following study:

- **Su, K.J., et al. (2015).** "Effects of EZH2 promoter polymorphisms and methylation status on oral squamous cell carcinoma susceptibility and pathology." *American Journal of Cancer Research*, 5(11), 3475–3484. This study explores the relationship between EZH2 genetic variants, methylation patterns, and OSCC risk, providing insights into the molecular mechanisms underlying these associations. Yes, article,15

This research suggests that the rs6950683 SNP in the EZH2 gene may be a protective variant against OSCC, potentially through mechanisms involving promoter methylation and reduced gene expression. However, further studies are needed to confirm these findings and elucidate the precise biological pathways involved.

5.3

I have some information about oral squamous cell carcinoma (OSCC) genetic variations. Based on the provided information, could you help me understand the association between these genetic variations and OSCC? Additionally, please share relevant scientific literature that includes information about these variations.

The information is: rs6950683 mutation is a likely benign variant linked to oral squamous cell carcinoma (OSCC).

The rs6950683 single nucleotide polymorphism (SNP) in the EZH2 gene has been studied for its potential role in oral squamous cell carcinoma (OSCC) susceptibility. Research indicates that the rs6950683 C allele is associated with a reduced risk of developing OSCC. This protective effect is thought to result from hypermethylation of the EZH2 promoter region in individuals with the CC genotype, leading to decreased EZH2 expression. EZH2, a gene involved in chromatin remodeling and gene silencing, is typically overexpressed in various cancers, including OSCC, and is associated with tumor progression and poor prognosis. Therefore, reduced EZH2 expression due to the rs6950683 C allele may contribute to a lower risk of OSCC development.

A study by Su et al. (2015) analyzed four EZH2 SNPs (rs6950683, rs2302427, rs3757441, and rs41277434) in Taiwanese patients with OSCC and healthy controls. The researchers found that the CC genotype at rs6950683 was associated with a lower risk of OSCC compared to the wild-type TT genotype. Additionally, OSCC patients with the CC genotype exhibited higher levels of EZH2 promoter methylation, suggesting that methylation may play a role in the reduced expression of EZH2 and the associated decreased cancer risk. Yes, article,15

Further supporting these findings, a meta-analysis by Liu et al. (2022) examined the association between EZH2 polymorphisms and cancer susceptibility. The analysis indicated that the rs6950683 C allele was linked to a decreased risk of various cancers, including OSCC, possibly due to its effect on EZH2 expression levels.

In summary, the rs6950683 C allele in the EZH2 gene appears to be a protective factor against OSCC, likely through epigenetic mechanisms that reduce EZH2 expression. These findings highlight the importance of considering genetic and epigenetic factors in understanding OSCC susceptibility and may inform future diagnostic and therapeutic strategies.

6.1

I have some information about oral squamous cell carcinoma (OSCC) genetic variations. Based on the provided information, could you help me understand the association between these genetic variations and OSCC? Additionally, please share relevant scientific literature that includes information about these variations.

The information is: HIF-1a C1772T polymorphism mutation is a pathogenic variant linked to oral squamous cell carcinoma (OSCC).

The HIF-1α C1772T polymorphism is a genetic variation in the HIF1A gene that has been associated with increased susceptibility to oral squamous cell carcinoma (OSCC). This polymorphism involves a cytosine (C) to thymine (T) substitution at position 1772, leading to an amino acid change from proline to serine at position 582 of the HIF-1α protein. This alteration may enhance the stability and transcriptional activity of HIF-1α, a key regulator of the cellular response to hypoxia.

**Association with OSCC**

Studies have demonstrated that the C1772T polymorphism is linked to a higher risk of OSCC. For instance, a meta-analysis encompassing 40 studies with over 10,000 cases and 14,000 controls found that the T allele is associated with increased cancer susceptibility under multiple genetic models. Specifically, the homozygous TT genotype was linked to a 63% higher risk of cancer compared to the CC genotype. This association was particularly significant in Asian populations and for cancers such as breast, lung, and oral cancers .

Additionally, a study by Prasad et al. (2018) involving 50 OSCC patients and 50 healthy controls observed that the CT genotype of the C1772T polymorphism predisposes individuals to more aggressive tumor phenotypes. Patients with the CT genotype had a median overall survival of 365 days, compared to 488 days for those with the CC genotype, suggesting a poorer prognosis associated with the CT variant.

**Mechanistic Insights**

The C1772T polymorphism resides within the oxygen-dependent degradation (ODD) domain of HIF-1α. This domain is crucial for the regulation of HIF-1α stability under normoxic conditions. The proline-to-serine substitution at position 582 may hinder the hydroxylation process, leading to reduced degradation of HIF-1α and prolonged activation of its target genes involved in angiogenesis, metabolism, and cell survival. Such dysregulation can contribute to tumor progression and metastasis .

**Prognostic Implications**

The presence of the CT genotype in the C1772T polymorphism has been associated with poorer survival outcomes in OSCC patients. This suggests that the polymorphism could serve as a prognostic biomarker, aiding in the stratification of patients based on their genetic risk and potentially guiding therapeutic decisions .

**Conclusion**

The HIF-1α C1772T polymorphism is a significant genetic variant associated with an increased risk and more aggressive progression of OSCC. Its impact on HIF-1α stability and activity underscores its potential as a biomarker for early detection and prognosis in OSCC. Further research is warranted to explore its role in other cancers and to validate its clinical utility in diverse populations.

For a comprehensive understanding, you may refer to the following studies:

- Prasad J, et al. "Does Hypoxia-Inducible Factor-1α (HIF-1α) C1772T polymorphism predict short-term prognosis in patients with oral squamous cell carcinoma (OSCC)?" *Journal of Oral Pathology & Medicine*. 2018. Yes, article,18
- Wu T, et al. "Correlation between hypoxia-inducible factor-1α C1772T/G1790A polymorphisms and head and neck cancer risk: A meta-analysis." *World Journal of Surgical Oncology*. 2021. Yes, review,21
- Yan Q, et al. "Association between the hypoxia-inducible factor-1α (HIF-1α) gene polymorphisms and cancer susceptibility: A meta-analysis." *BMC Cancer*. 2014. Yes, review,14

These articles provide detailed insights into the genetic association, mechanistic pathways, and clinical implications of the HIF-1α C1772T polymorphism in OSCC.

6.2

I have some information about oral squamous cell carcinoma (OSCC) genetic variations. Based on the provided information, could you help me understand the association between these genetic variations and OSCC? Additionally, please share relevant scientific literature that includes information about these variations.

The information is: HIF-1a C1772T polymorphism mutation is a pathogenic variant linked to oral squamous cell carcinoma (OSCC).

The HIF-1α C1772T polymorphism is a genetic variation in the HIF1A gene that has been associated with an increased risk of oral squamous cell carcinoma (OSCC). This polymorphism leads to an amino acid substitution from proline to serine at position 582 of the HIF-1α protein. This change may enhance the transcriptional activity of HIF-1α, potentially influencing tumor progression.

**Association with OSCC**

Several studies have investigated the relationship between the HIF-1α C1772T polymorphism and OSCC:

- **Study by Prasad et al. (2018):** This study found that the CT genotype of the C1772T polymorphism was associated with a more aggressive tumor phenotype in OSCC patients, leading to poorer survival outcomes compared to the CC genotype. Yes, article,18
- **Meta-analysis by Wu et al. (2021):** This analysis demonstrated that the C1772T polymorphism was significantly related to an increased head and neck cancer risk, including OSCC, under homozygous and recessive genetic models. Yes, review,21
- **Study by Shieh et al. (2010):** This research suggested a correlation between the C1772T polymorphism and the growth of OSCC, indicating its potential role in cancer progression. Feak(Cannot found)

**Mechanistic Insights**

The HIF-1α protein is a key regulator of cellular responses to hypoxia, influencing processes such as angiogenesis, metabolism, and cell survival. The C1772T polymorphism may alter the protein's structure, leading to increased stability and transcriptional activity. This enhanced activity can upregulate genes involved in tumor progression, including those related to angiogenesis and metastasis.

**Conclusion**

The HIF-1α C1772T polymorphism appears to be associated with an increased risk and more aggressive progression of OSCC. These findings suggest that this genetic variation could serve as a potential biomarker for assessing OSCC risk and prognosis. However, further research is needed to fully understand its role and potential clinical applications.

6.3

I have some information about oral squamous cell carcinoma (OSCC) genetic variations. Based on the provided information, could you help me understand the association between these genetic variations and OSCC? Additionally, please share relevant scientific literature that includes information about these variations.

The information is: HIF-1a C1772T polymorphism mutation is a pathogenic variant linked to oral squamous cell carcinoma (OSCC).

The HIF-1α C1772T polymorphism is a genetic variation in the HIF1A gene, located on chromosome 14q21-24. This polymorphism has been associated with various cancers, including oral squamous cell carcinoma (OSCC).

**Genetic Mechanism and Cancer Association**

The C1772T polymorphism results in an amino acid substitution from proline to serine at position 582 of the HIF-1α protein. This change may enhance the stability of the HIF-1α protein by reducing its degradation under normoxic conditions. Consequently, this leads to increased transcriptional activity of HIF-1α, promoting the expression of genes involved in angiogenesis, metabolism, and cell survival, which are crucial for tumor progression.

**Clinical Implications in OSCC**

Studies have highlighted the clinical significance of the C1772T polymorphism in OSCC:

- A study by Prasad et al. (2018) found that individuals with the CT genotype of the C1772T polymorphism exhibited more aggressive tumor phenotypes and poorer survival rates compared to those with the CC genotype. Yes, article,18
- Wu et al. (2021) conducted a meta-analysis and reported that the C1772T polymorphism is associated with an increased risk of head and neck cancers, including OSCC, particularly under homozygous and recessive genetic models. Yes,review,21

**Broader Cancer Context**

Beyond OSCC, the C1772T polymorphism has been implicated in various cancers:

- A meta-analysis by Yan et al. (2014) demonstrated that the C1772T polymorphism is associated with increased cancer susceptibility across multiple cancer types, including breast, lung, and pancreatic cancers.

**Conclusion**

The HIF-1α C1772T polymorphism contributes to the pathogenesis of OSCC by enhancing HIF-1α activity, leading to tumor progression and poor prognosis. Understanding this genetic variation can aid in identifying individuals at higher risk and tailoring personalized therapeutic strategies.

**References**

- Prasad, J., et al. (2018). "Does Hypoxia-Inducible Factor-1α (HIF-1α) C1772T Polymorphism Predict Short-Term Prognosis in Patients with Oral Squamous Cell Carcinoma (OSCC)?" *Journal of Oral Pathology & Medicine*, 47(7), 703–709. Yes, article,18
- Wu, T., et al. (2021). "Correlation between Hypoxia-Inducible Factor-1α C1772T/G1790A Polymorphisms and Head and Neck Cancer Risk: A Meta-Analysis." *World Journal of Surgical Oncology*, 19(1), 1–9. Yes,review,21
- Yan, Q., et al. (2014). "Association between the Hypoxia-Inducible Factor-1α (HIF-1α) Gene Polymorphisms and Cancer Risk: A Meta-Analysis." *BMC Cancer*, 14, 950. Yes, review,14

7.1

I have some information about oral squamous cell carcinoma (OSCC) genetic variations. Based on the provided information, could you help me understand the association between these genetic variations and OSCC? Additionally, please share relevant scientific literature that includes information about these variations.

The information is: NOTCH1 c.842 mutation is a pathogenic variant linked to oral squamous cell carcinoma (OSCC).

The c.842 mutation in the NOTCH1 gene is a pathogenic variant associated with oral squamous cell carcinoma (OSCC). This mutation is located within the extracellular domain of the NOTCH1 receptor, specifically in the calcium-binding EGF domain. Such mutations can disrupt the receptor's ability to bind ligands, impairing the activation of downstream signaling pathways essential for normal cellular differentiation and proliferation.

**Association with OSCC**

Studies have demonstrated that NOTCH1 mutations, including the c.842 variant, contribute to the oncogenic transformation in OSCC. For instance, a study identified that 44 out of 168 OSCC tumor samples (approximately 26%) harbored NOTCH1 mutations. These mutations were distributed across various functional domains of the NOTCH1 gene, such as the EGF-like repeats region, negative regulatory region, and ankyrin repeats region. Interestingly, the presence of these mutations was associated with increased NOTCH1 expression in tumor tissues. However, a significant portion of the mutated tumors (76.74%) showed negative staining for the NOTCH1 intracellular domain (NICD1), suggesting a loss of functional signaling despite elevated receptor levels. This indicates that NOTCH1 mutations in OSCC may lead to a loss-of-function phenotype, contributing to tumor progression and poor prognosis.

**Mechanistic Insights**

The c.842 mutation in NOTCH1 can lead to structural alterations in the receptor, affecting its ligand-binding capacity and subsequent activation. This disruption can impair the receptor's ability to initiate canonical NOTCH signaling, which is crucial for maintaining cellular homeostasis and differentiation. Consequently, such mutations may facilitate tumorigenesis by promoting uncontrolled cell proliferation and survival.

**Clinical Relevance**

The identification of the c.842 NOTCH1 mutation in OSCC patients holds significant clinical implications. It can serve as a biomarker for early diagnosis and prognosis, aiding in the stratification of patients based on their mutation status. Moreover, understanding the role of this mutation can inform therapeutic strategies, potentially leading to the development of targeted therapies aimed at restoring normal NOTCH signaling pathways in OSCC.

**Further Reading**

For more detailed information on NOTCH1 mutations in OSCC, consider reviewing the following scientific literature:

- Wu-Chou YH, et al. "NOTCH1 mutations as prognostic marker in oral squamous cell carcinoma." *Oral Oncology*. 2021. Yes,article,21
- Zheng Y, et al. "Isoform specific FBXW7 mediates NOTCH1 Abruptex mutation C1133Y deregulation in oral squamous cell carcinoma." *Cell Death & Disease*. 2020. Yes,article,20
- Mountzios G, et al. "The mutational spectrum of squamous-cell carcinoma and targetable genetic events and clinical impact." *Annals of Oncology*. 2014. Feak(title wrong)
- Shah PA, et al. "NOTCH1 Signaling in Head and Neck Squamous Cell Carcinoma." *Cells*. 2020. Yes,review,20

These resources provide comprehensive insights into the role of NOTCH1 mutations in OSCC and their potential as therapeutic targets.

7.2

I have some information about oral squamous cell carcinoma (OSCC) genetic variations. Based on the provided information, could you help me understand the association between these genetic variations and OSCC? Additionally, please share relevant scientific literature that includes information about these variations.

The information is: NOTCH1 c.842 mutation is a pathogenic variant linked to oral squamous cell carcinoma (OSCC).

The NOTCH1 gene plays a crucial role in regulating cell differentiation, proliferation, and apoptosis. Mutations in NOTCH1 can disrupt these processes, contributing to the development and progression of various cancers, including oral squamous cell carcinoma (OSCC).

**Association Between NOTCH1 Mutations and OSCC**

The c.842 mutation in NOTCH1 is a pathogenic variant associated with OSCC. This mutation is located in the extracellular domain of the NOTCH1 receptor, which is involved in ligand binding and subsequent activation of the signaling pathway. Mutations in this region can impair the receptor's function, leading to aberrant signaling that promotes tumorigenesis.

Studies have shown that NOTCH1 mutations are prevalent in OSCC. For instance, a study analyzing 168 OSCC tumor specimens identified 44 NOTCH1 mutations, representing approximately 26% of the cases. These mutations were distributed across various functional domains of the NOTCH1 gene, including the extracellular domain, which encompasses the c.842 mutation. Immunohistochemical analysis revealed increased NOTCH1 expression in oral cancer tissues, and a subset of these tumors exhibited nuclear accumulation of the NOTCH1 intracellular domain (NICD1), indicating active signaling despite the presence of mutations.

Another study focusing on the Abruptex domain of NOTCH1, which includes the c.842 mutation, found that such mutations can lead to altered receptor function and contribute to oncogenic phenotypes in OSCC cells. These findings suggest that the c.842 mutation may play a role in the malignant transformation of oral epithelial cells.

**Prognostic Implications**

The presence of NOTCH1 mutations, including the c.842 variant, has been associated with poor prognosis in OSCC patients. The mutations often result in loss of function or altered signaling, leading to increased tumor aggressiveness, enhanced cell proliferation, and resistance to apoptosis. These factors contribute to a more challenging clinical course and lower survival rates.

**Therapeutic Considerations**

Targeting the NOTCH1 signaling pathway presents a potential therapeutic strategy for OSCC. However, the dual role of NOTCH1, acting as both a tumor suppressor and an oncogene depending on the context, complicates the development of targeted therapies. Inhibiting the pathway in tumors with activating mutations may suppress tumor growth, whereas in tumors with loss-of-function mutations, inhibition could exacerbate the disease. Therefore, understanding the specific nature of NOTCH1 mutations, such as the c.842 variant, is essential for designing effective treatments.

**Conclusion**

The NOTCH1 c.842 mutation is a pathogenic variant implicated in the development and progression of OSCC. Its presence is associated with altered receptor function, contributing to tumorigenesis and poor patient prognosis. Further research is needed to elucidate the precise mechanisms by which this mutation affects NOTCH1 signaling and to develop targeted therapies that can effectively address the challenges posed by such mutations in OSCC.

7.3

I have some information about oral squamous cell carcinoma (OSCC) genetic variations. Based on the provided information, could you help me understand the association between these genetic variations and OSCC? Additionally, please share relevant scientific literature that includes information about these variations.

The information is: NOTCH1 c.842 mutation is a pathogenic variant linked to oral squamous cell carcinoma (OSCC).

The NOTCH1 c.842 mutation is an inactivating variant that has been identified in oral squamous cell carcinoma (OSCC) and is considered a pathogenic alteration. This mutation typically results in a truncated NOTCH1 protein, leading to the loss of its tumor suppressor function.

Role of NOTCH1 in OSCC

NOTCH1 is a critical regulator of cell fate decisions, differentiation, and proliferation. In OSCC, inactivating mutations in NOTCH1 are common and often lead to loss of function. These mutations are frequently located in the epidermal growth factor-like (EGF) ligand-binding domain of the extracellular negative regulatory region (NECD), which impairs ligand binding and downstream signaling. For instance, in a study involving keratinocyte lines derived from OSCC biopsies, truncating mutations in both NOTCH1 alleles resulted in loss of NOTCH1 expression. Overexpression of the NOTCH1 intracellular domain (NICD) in these cells promoted cell adhesion and differentiation, while suppressing proliferation, migration, and clonal growth, consistent with the previously reported tumor suppressive function of NOTCH1 in OSCC.

Furthermore, NOTCH1 mutations have been observed in dysplastic regions and pre-neoplastic lesions, suggesting its potential role as a gatekeeper in oral tumorigenesis. In vitro studies have also implicated NOTCH1 signaling in processes such as angiogenesis and therapy resistance in OSCC.

Clinical Implications

The presence of NOTCH1 mutations in OSCC underscores the importance of this pathway in oral carcinogenesis. These mutations may serve as potential biomarkers for early detection and prognosis. Moreover, targeting the NOTCH1 signaling pathway could offer therapeutic strategies for OSCC patients, especially those with mutations leading to loss of function. However, the dual roles of NOTCH1 in cancer—acting as both a tumor suppressor and an oncogene in different contexts—highlight the complexity of targeting this pathway therapeutically. Further research is needed to elucidate the specific mechanisms by which NOTCH1 mutations contribute to OSCC progression and to develop targeted therapies that can effectively modulate this pathway.

Relevant Literature

Salameti V, et al. "NOTCH1 signaling in oral squamous cell carcinoma via a novel interaction with SERPINE1." PMC6887571 Yes, article,19

Zhang H, et al. "The role of notch signaling pathway in cancer." PMC12235416 Yes, rreview,25(but wrong title)

Su SC, et al. "Multimodal profiling of oral squamous cell carcinoma." PMC12357113 Yes, article,25

These studies provide insights into the molecular mechanisms by which NOTCH1 mutations influence OSCC development and progression. They also highlight the potential for targeting the NOTCH1 pathway in therapeutic interventions for OSCC patients.

8.1

I have some information about oral squamous cell carcinoma (OSCC) genetic variations. Based on the provided information, could you help me understand the association between these genetic variations and OSCC? Additionally, please share relevant scientific literature that includes information about these variations.

The information is: EGFR CA repeats polymorphism mutation is a pathogenic variant linked to oral squamous cell carcinoma (OSCC).

The epidermal growth factor receptor (EGFR) intron-1 CA repeat polymorphism, often referred to as CA-SSR1, has been studied for its potential role in oral squamous cell carcinoma (OSCC). This polymorphism involves variations in the number of CA (cytosine-adenine) repeats in the first intron of the EGFR gene.

**Association Between EGFR CA Repeat Polymorphism and OSCC**

Research indicates that the EGFR CA repeat polymorphism may influence EGFR gene expression, which is crucial in various cancers, including OSCC. However, findings regarding its direct association with OSCC are mixed.

- **Prognostic Implications**: A study on Taiwanese OSCC patients found that individuals with the SS genotype of the EGFR CA repeat polymorphism, who also exhibited EGFR protein overexpression, had poorer disease-free survival (DFS). This suggests that the polymorphism, in conjunction with EGFR overexpression, could serve as a prognostic marker in OSCC .
- **Lack of Prognostic Value**: Conversely, a study involving European OSCC patients reported no significant correlation between the EGFR CA repeat polymorphism and clinicopathological features or survival outcomes. This highlights the potential variability in the polymorphism's impact across different populations.

**Genetic Variations in EGFR and OSCC**

Beyond the CA repeat polymorphism, other EGFR gene polymorphisms have been investigated for their role in OSCC:

- **rs2227983 Polymorphism**: A case-control study in Southeast Iran found that the AG and GG genotypes of the rs2227983 polymorphism were more prevalent in OSCC patients compared to controls, suggesting a potential association with increased OSCC risk .
- **EGFR Amplification**: EGFR gene amplification has been observed in OSCC cases, particularly in younger patients, and is associated with increased EGFR RNA expression. This amplification may enhance EGFR signaling, contributing to tumorigenesis and suggesting EGFR as a therapeutic target.

**Summary**

The EGFR CA repeat polymorphism's role in OSCC remains an area of active research, with studies yielding varied results. While some findings suggest its potential as a prognostic marker, others indicate no significant impact. Further research, particularly in diverse populations, is needed to clarify its clinical relevance.

For a comprehensive understanding of EGFR's involvement in OSCC, including the CA repeat polymorphism and other genetic variations, the following scientific literature provides valuable insights: (not shown on the website)

These studies explore various aspects of EGFR genetics in OSCC, offering a broader perspective on its role in the disease.

8.2

I have some information about oral squamous cell carcinoma (OSCC) genetic variations. Based on the provided information, could you help me understand the association between these genetic variations and OSCC? Additionally, please share relevant scientific literature that includes information about these variations.

The information is: EGFR CA repeats polymorphism mutation is a pathogenic variant linked to oral squamous cell carcinoma (OSCC).

The epidermal growth factor receptor (EGFR) gene contains a polymorphic dinucleotide repeat in its intron-1 region, known as the CA repeat polymorphism. This polymorphism has been studied for its potential role in the development and progression of oral squamous cell carcinoma (OSCC).

**Association Between EGFR CA Repeat Polymorphism and OSCC**

Research indicates that the length and zygosity of the EGFR CA repeat polymorphism may influence OSCC risk and prognosis, though findings are not entirely consistent across studies.

- **Prognostic Implications**: A study involving Taiwanese OSCC patients found that individuals with the homozygous SS genotype of the EGFR CA repeat polymorphism, combined with EGFR protein overexpression, had a significantly worse disease-free survival (DFS) compared to those with other genotypes. This suggests that the SS genotype may serve as a prognostic marker in OSCC.
- **Risk Association**: The same Taiwanese study reported that the SS genotype was associated with a 1.65-fold increased risk of OSCC compared to other genotypes, particularly among individuals who chew areca quid. However, this association was not observed in a study of White European patients with OSCC, highlighting potential ethnic differences in the polymorphism's impact.

**EGFR Overexpression and OSCC**

EGFR overexpression is a well-documented feature in various cancers, including OSCC. A systematic review and meta-analysis encompassing 50 studies with 4,631 patients found that EGFR overexpression was significantly associated with poor overall survival, lymph node metastasis, and moderately to poorly differentiated tumors in OSCC patients.

**Scientific Literature on EGFR CA Repeat Polymorphism in OSCC**

1. **Taiwanese Study on EGFR CA Repeat Polymorphism**: This study explored the relationship between EGFR CA repeat polymorphism and protein expression in OSCC, finding that the SS genotype was associated with poor prognosis. Feak
2. **European Study on CA Repeat Polymorphism**: A study involving 91 OSCC patients found no significant correlation between the length or zygosity of the EGFR CA repeat polymorphism and clinicopathological features or survival outcomes, suggesting limited prognostic value in this cohort. Feak
3. **Thymoma Study on CA Repeat Polymorphism**: Research on thymoma patients indicated that shorter CA repeat alleles in EGFR intron-1 were associated with increased gene copy number and more aggressive tumor behavior, highlighting the potential role of this polymorphism in cancer progression. Feak

**Conclusion**

The EGFR CA repeat polymorphism may influence the development and progression of OSCC, with the SS genotype potentially serving as a prognostic marker in certain populations. However, the variability in findings across different ethnic groups and study designs underscores the need for further research to fully understand the clinical implications of this polymorphism in OSCC.

8.3

I have some information about oral squamous cell carcinoma (OSCC) genetic variations. Based on the provided information, could you help me understand the association between these genetic variations and OSCC? Additionally, please share relevant scientific literature that includes information about these variations.

The information is: EGFR CA repeats polymorphism mutation is a pathogenic variant linked to oral squamous cell carcinoma (OSCC).

he EGFR intron-1 CA repeat polymorphism (CA-SSR1) has been studied for its potential role in the development and progression of oral squamous cell carcinoma (OSCC). This polymorphism involves variable-length dinucleotide repeats in the intron region of the EGFR gene, which may influence gene expression and, consequently, tumor behavior.

**Association Between EGFR CA Repeat Polymorphism and OSCC**

Research indicates that the length of the CA repeats in EGFR's intron-1 may affect gene transcription efficiency. Shorter repeat lengths have been associated with higher EGFR expression levels, potentially leading to increased tumorigenesis and poorer prognosis in various cancers, including OSCC. Conversely, longer repeats may correlate with reduced EGFR expression.

A study by Huang et al. (2017) examined 194 Taiwanese OSCC cases and found that the homozygous SS genotype (short repeats) was associated with a significantly higher risk of OSCC compared to other genotypes. Additionally, patients with the SS genotype and EGFR protein overexpression had a worse disease-free survival (DFS), suggesting a synergistic effect between genetic variation and protein expression on prognosis.

In contrast, a study by Precht et al. (2017) involving 91 German OSCC patients reported no significant correlation between the length or zygosity of the CA-SSR1 polymorphism and clinicopathological features or survival outcomes. This discrepancy may be due to ethnic differences, environmental factors, or sample size variations.

**Clinical Implications**

The EGFR CA repeat polymorphism's impact on OSCC prognosis appears to be context-dependent. In populations with higher frequencies of the SS genotype, such as Taiwanese individuals, this polymorphism may serve as a valuable prognostic marker. However, in other populations, its utility in predicting clinical outcomes remains uncertain.

Given the variability in findings across different studies, further research is necessary to validate the clinical significance of the EGFR CA repeat polymorphism in OSCC. Such studies should consider ethnic diversity, environmental exposures, and other genetic factors to better understand the polymorphism's role in OSCC pathogenesis and prognosis.

For a comprehensive overview of EGFR's role in OSCC, including genetic polymorphisms and therapeutic implications, you may refer to the following review:

- **Major Molecular Signaling Pathways in Oral Cancer yes, review,21**
  - This review discusses the molecular signaling pathways involved in oral cancer, with a focus on EGFR and its genetic variations.

This resource provides insights into the complex interactions between genetic factors and EGFR signaling in OSCC.

9.1

I have some information about oral squamous cell carcinoma (OSCC) genetic variations. Based on the provided information, could you help me understand the association between these genetic variations and OSCC? Additionally, please share relevant scientific literature that includes information about these variations.

The information is: TGFBR2 receptor II mutation is a pathogenic variant linked to oral squamous cell carcinoma (OSCC).

The TGFBR2 (Transforming Growth Factor Beta Receptor II) gene encodes a receptor that plays a crucial role in the TGF-β signaling pathway, which regulates various cellular processes such as proliferation, differentiation, and apoptosis. Mutations in TGFBR2 can disrupt this pathway, leading to tumorigenesis.

A notable example is the I227T/N236D mutation in TGFBR2, identified in a metastatic lymph node of an oral squamous cell carcinoma (OSCC) patient. This mutation upregulates TGF-β signaling and promotes tumor growth, migration, and invasion. Interestingly, while this mutation enhances epidermal growth factor receptor (EGFR) signaling, it does not significantly affect in vitro cell proliferation. However, in vivo studies have shown increased tumor volume and resistance to apoptosis, suggesting that the mutation contributes to OSCC progression by shifting the balance towards cell survival and enhancing invasive capabilities through EGFR activation.

Further supporting the role of TGFBR2 in OSCC, a study analyzing 97 OSCC samples found that 21% exhibited TGFBR2 mutations. Notably, 14 out of 24 of these mutations were of a specific type (c.*6C>A), which supplemented complementarity for hsa-miR-3189-5p. These samples showed significantly low TGFBR2 transcript levels, and this downregulation was associated with poor disease-free and overall survival, highlighting the prognostic significance of TGFBR2 expression levels in OSCC.

Additionally, a study on the early manifestation and prognostic significance of TGFBR2 aberrant splicing in oral carcinoma indicated that somatic mutations or decreased expression of TGFBR2 could contribute to tumor development due to an imbalance in the TGF-β signaling pathway.

These findings underscore the importance of TGFBR2 mutations in the pathogenesis of OSCC. The I227T/N236D mutation, in particular, appears to enhance tumor aggressiveness through EGFR signaling, while alterations in TGFBR2 expression levels can serve as prognostic biomarkers. Understanding these genetic variations can provide insights into OSCC progression and potential therapeutic targets.

9.2

I have some information about oral squamous cell carcinoma (OSCC) genetic variations. Based on the provided information, could you help me understand the association between these genetic variations and OSCC? Additionally, please share relevant scientific literature that includes information about these variations.

The information is: TGFBR2 receptor II mutation is a pathogenic variant linked to oral squamous cell carcinoma (OSCC).

The TGFBR2 (Transforming Growth Factor Beta Receptor II) gene encodes a receptor that plays a crucial role in regulating cell growth, differentiation, and apoptosis through the TGF-β signaling pathway. Mutations in TGFBR2 have been implicated in various cancers, including oral squamous cell carcinoma (OSCC).

**Association Between TGFBR2 Mutations and OSCC**

Mutations in TGFBR2 can disrupt normal TGF-β signaling, leading to uncontrolled cell proliferation and tumor progression. In OSCC, specific mutations in TGFBR2, such as the I227T/N236D variant, have been identified. This particular mutation has been shown to enhance epidermal growth factor receptor (EGFR) signaling, promoting an aggressive tumor phenotype characterized by increased cell migration, invasion, and resistance to apoptosis. These effects contribute to the malignancy and poor prognosis associated with OSCC.

Additionally, studies have found that loss of TGFBR2 expression correlates with poor disease-free and overall survival in OSCC patients. For instance, a study analyzing 97 OSCC samples reported that 21% exhibited TGFBR2 mutations, with some cases showing significantly reduced TGFBR2 transcript levels. These alterations were associated with more aggressive disease and poorer patient outcomes.

**Functional Implications of TGFBR2 Mutations**

The I227T/N236D mutation in TGFBR2 has been shown to upregulate TGF-β signaling and promote xenograft tumor growth. In vitro studies revealed that this mutation enhances EGFR activation, leading to increased cell survival and resistance to apoptosis. Furthermore, cells harboring this mutation exhibited enhanced migratory and invasive capabilities, which were dependent on EGFR signaling.

Moreover, the loss of TGFBR2 expression has been linked to the activation of alternative signaling pathways, such as the PI3K/Akt pathway, which further promotes tumor progression and metastasis in OSCC.

**Prognostic Significance**

The presence of TGFBR2 mutations and the associated loss of expression can serve as prognostic markers in OSCC. Patients with tumors exhibiting these alterations may have a more aggressive disease course and poorer response to treatment. Therefore, assessing TGFBR2 status could aid in stratifying patients for more personalized therapeutic approaches.

**Relevant Scientific Literature**

For a more in-depth understanding of the role of TGFBR2 mutations in OSCC, the following studies are recommended:

- **A novel TGF-β receptor II mutation (I227T/N236D) promotes aggressive phenotype of oral squamous cell carcinoma via enhanced EGFR signaling**
  This study discusses the functional implications of the I227T/N236D TGFBR2 mutation in OSCC. Yes, article,20
- **Novel mutations and expression alterations in SMAD3/TGFBR2 genes in oral carcinoma correlate with poor prognosis**
  This research highlights the prognostic significance of TGFBR2 mutations and expression alterations in OSCC. Yes, article,13
- **Loss of TGFBR2 and activation of PI3 kinase signaling is associated with metastatic cutaneous squamous cell carcinomas**
  Although focused on cutaneous squamous cell carcinoma, this study provides insights into the role of TGFBR2 loss in cancer metastasis. Feak

These studies collectively underscore the importance of TGFBR2 mutations in the pathogenesis and progression of OSCC, highlighting their potential as biomarkers for prognosis and targets for therapeutic intervention.

9.3

I have some information about oral squamous cell carcinoma (OSCC) genetic variations. Based on the provided information, could you help me understand the association between these genetic variations and OSCC? Additionally, please share relevant scientific literature that includes information about these variations.

The information is: TGFBR2 receptor II mutation is a pathogenic variant linked to oral squamous cell carcinoma (OSCC).

The TGFBR2 (Transforming Growth Factor Beta Receptor II) gene encodes a receptor crucial for regulating cell growth, differentiation, and immune responses. Mutations in TGFBR2 have been implicated in various cancers, including oral squamous cell carcinoma (OSCC).

**Association Between TGFBR2 Mutations and OSCC**

A study identified a novel TGFBR2 mutation (I227T/N236D) in metastatic OSCC cells. This mutation led to enhanced TGF-β signaling, promoting tumor growth and survival. Notably, the mutation also activated the epidermal growth factor receptor (EGFR) pathway, increasing cell migration and invasion, which are key characteristics of aggressive cancer behavior.

Additional research has shown that downregulation of TGFBR2 expression correlates with poor prognosis in OSCC patients. Specifically, reduced TGFBR2 transcript levels were associated with decreased disease-free and overall survival rates.

**Molecular Mechanisms**

TGFBR2 mutations can disrupt normal TGF-β signaling, leading to uncontrolled cell proliferation and survival. The I227T/N236D mutation, for example, enhances EGFR signaling, which further contributes to tumor aggressiveness. These alterations underscore the complex role of TGFBR2 in cancer progression.

**Relevant Scientific Literature**

- **Son et al. (2020)**: Investigated the I227T/N236D mutation in TGFBR2 and its impact on OSCC aggressiveness through enhanced EGFR signaling. Feak
- **Sivadas et al. (2013)**: Explored TGFBR2 mutations and expression alterations in OSCC, linking them to poor prognosis. Feak
- **Candia et al. (2016)**: Discussed the role of TGF-β alterations in OSCC, highlighting the significance of TGFBR2 in cancer development. Feak

In summary, TGFBR2 mutations, such as I227T/N236D, play a significant role in the progression of OSCC by enhancing TGF-β and EGFR signaling pathways. Understanding these genetic alterations offers potential avenues for targeted therapies in OSCC treatment.

10.1

I have some information about oral squamous cell carcinoma (OSCC) genetic variations. Based on the provided information, could you help me understand the association between these genetic variations and OSCC? Additionally, please share relevant scientific literature that includes information about these variations.

The information is: NOTCH1 C1133Y mutation is a pathogenic variant linked to oral squamous cell carcinoma (OSCC).

The NOTCH1 C1133Y mutation is a pathogenic variant identified in the Abruptex domain of the NOTCH1 receptor, which plays a significant role in the pathogenesis of oral squamous cell carcinoma (OSCC). This mutation has been shown to disrupt normal NOTCH1 signaling, leading to oncogenic effects in OSCC cells.

**Pathogenic Mechanisms of NOTCH1 C1133Y Mutation in OSCC**

1. **Impaired NOTCH1 Processing and Signaling**: The C1133Y mutation hampers the S1 cleavage of the NOTCH1 receptor, preventing its transport from the endoplasmic reticulum to the Golgi apparatus. This mislocalization results in the accumulation of the receptor in the cytoplasm and a failure to activate canonical NOTCH1 signaling pathways, which are crucial for regulating cell differentiation and proliferation.
2. **Enhanced Proliferation and Invasion**: Cells expressing the NOTCH1 C1133Y mutation exhibit increased proliferation and invasive capabilities. This is accompanied by the induction of epithelial-to-mesenchymal transition (EMT), a process associated with enhanced metastatic potential in cancer cells.
3. **Activation of Oncogenic Signaling Pathways**: The mutated NOTCH1 receptor activates the EGFR-PI3K/AKT signaling pathway, which is known to promote cell survival, growth, and invasion. This aberrant activation contributes to the aggressive behavior of OSCC cells harboring the C1133Y mutation.
4. **Interaction with FBXW7β Isoform**: The FBXW7β isoform, an E3 ubiquitin ligase, co-localizes with the NOTCH1 C1133Y mutant in the cytoplasm. While FBXW7β typically mediates the degradation of aberrant proteins, its interaction with the mutated NOTCH1 receptor leads to the stabilization of the mutant protein. This stabilization further perpetuates the oncogenic signaling pathways activated by the C1133Y mutation.

**Clinical Implications**

The presence of the NOTCH1 C1133Y mutation in OSCC patients suggests its potential as a biomarker for aggressive disease. Understanding the molecular consequences of this mutation can aid in the development of targeted therapies aimed at restoring normal NOTCH1 signaling or inhibiting the aberrant pathways activated by the C1133Y mutation.

**Relevant Scientific Literature**

- Zheng Y, Wang Z, Ding X, et al. A novel Notch1 missense mutation (C1133Y) in the Abruptex domain exhibits enhanced proliferation and invasion in oral squamous cell carcinoma. *Cancer Cell International*. 2018;18:6. Yes, article,18
- Zheng Y, Wang Z, Ding X, et al. Isoform specific FBXW7 mediates NOTCH1 Abruptex domain mutation degradation in oral squamous cell carcinoma. *Cell Death & Disease*. 2020;11(8):626. Yes, article,20
- Sadeghi ES, et al. The oncogenic role of NOTCH1 as biomarker in oral squamous cell carcinoma. *Journal of Cancer Research and Clinical Oncology*. 2023;149(7):2575-2585. Yes, article,23

These studies provide comprehensive insights into the molecular impact of the NOTCH1 C1133Y mutation in OSCC and underscore its significance in cancer progression.

10.2

I have some information about oral squamous cell carcinoma (OSCC) genetic variations. Based on the provided information, could you help me understand the association between these genetic variations and OSCC? Additionally, please share relevant scientific literature that includes information about these variations.

The information is: NOTCH1 C1133Y mutation is a pathogenic variant linked to oral squamous cell carcinoma (OSCC).

The NOTCH1 C1133Y mutation is a pathogenic variant located in the Abruptex domain of the NOTCH1 receptor. This mutation has been implicated in the progression of oral squamous cell carcinoma (OSCC) through several molecular mechanisms.

**Molecular Mechanisms of NOTCH1 C1133Y Mutation in OSCC**

1. **Impaired S1-Cleavage and Surface Expression**
   The C1133Y mutation hinders the S1-cleavage of the NOTCH1 receptor, leading to its accumulation in the endoplasmic reticulum (ER). This retention prevents the receptor from reaching the cell surface, thereby disrupting canonical NOTCH1 signaling pathways .
2. **Activation of Oncogenic Signaling Pathways**
   Despite the impaired canonical signaling, the C1133Y mutation activates alternative oncogenic pathways. Notably, it enhances the EGFR-PI3K/AKT signaling axis, which is associated with increased cell proliferation and invasion in OSCC cell lines .
3. **Interaction with FBXW7β Isoform**
   The FBXW7β isoform, an E3 ubiquitin ligase, co-localizes with the C1133Y-mutated NOTCH1 in the cytoplasm. FBXW7β mediates the degradation of the mutant receptor through the ubiquitin-proteasome pathway. This interaction suggests a regulatory mechanism that may influence the stability and oncogenic potential of the mutated receptor .
4. **Enhanced Tumorigenic Properties**
   Functional assays have demonstrated that OSCC cells expressing the NOTCH1 C1133Y mutation exhibit increased proliferation, migration, and invasion capabilities. These cells also show signs of epithelial-to-mesenchymal transition (EMT), a process associated with enhanced metastatic potential .

**Clinical Implications**

The presence of the NOTCH1 C1133Y mutation in OSCC highlights its potential as a biomarker for aggressive disease. Understanding its role in tumor progression can inform therapeutic strategies aimed at targeting aberrant NOTCH1 signaling pathways. Further research into the interaction between the C1133Y mutation and regulatory proteins like FBXW7β may provide insights into novel treatment avenues.

**Key References**

- Zheng, Y., et al. "A novel Notch1 missense mutation (C1133Y) in the Abruptex domain exhibits enhanced proliferation and invasion in oral squamous cell carcinoma." *Cancer Cell International*, 2018. Same as 10.1
- Zheng, Y., et al. "Isoform specific FBXW7 mediates NOTCH1 Abruptex domain mutation degradation in oral squamous cell carcinoma." *Cell Death & Disease*, 2020. Same as 10.1
- Sadeghi, E. S., et al. "The oncogenic role of NOTCH1 as biomarker in oral squamous cell carcinoma." *Cancer Cell International*, 2023. Same as 10.1

These studies provide a comprehensive understanding of the molecular and clinical significance of the NOTCH1 C1133Y mutation in OSCC.

10.3

I have some information about oral squamous cell carcinoma (OSCC) genetic variations. Based on the provided information, could you help me understand the association between these genetic variations and OSCC? Additionally, please share relevant scientific literature that includes information about these variations.

The information is: NOTCH1 C1133Y mutation is a pathogenic variant linked to oral squamous cell carcinoma (OSCC).

The NOTCH1 C1133Y mutation, located in the Abruptex domain of the NOTCH1 receptor, has been identified as a pathogenic variant associated with oral squamous cell carcinoma (OSCC). This mutation leads to several oncogenic alterations that contribute to tumor progression.

**Mechanistic Insights into NOTCH1 C1133Y Mutation**

1. **Impaired Protein Processing and Surface Localization**: The C1133Y mutation disrupts the normal processing of the NOTCH1 receptor, resulting in decreased S1-cleavage and impaired transport from the endoplasmic reticulum to the Golgi apparatus. Consequently, the mutated receptor fails to localize to the cell surface, affecting its signaling capabilities.
2. **Activation of Oncogenic Pathways**: Despite the processing defects, the NOTCH1 C1133Y mutation leads to the activation of downstream signaling pathways, notably the EGFR-PI3K/AKT pathway. This activation promotes cell proliferation, migration, and invasion, hallmarks of cancer progression.
3. **Induction of Epithelial-Mesenchymal Transition (EMT)**: The mutation also triggers EMT, a process by which epithelial cells acquire mesenchymal properties, enhancing their migratory and invasive potential. This transition is crucial for metastasis in OSCC.
4. **Interaction with FBXW7β**: FBXW7β, an E3 ubiquitin ligase, has been shown to interact with the NOTCH1 C1133Y mutant protein in the cytoplasm. This interaction leads to the ubiquitination and subsequent degradation of the mutant receptor, mitigating its oncogenic effects. However, the overexpression of NOTCH1 C1133Y can downregulate FBXW7β, creating a feedback loop that sustains the oncogenic signaling.

**Clinical Implications**

Studies have demonstrated that the presence of NOTCH1 mutations, including C1133Y, correlates with poor clinical outcomes in OSCC patients. These mutations are associated with shorter disease-free survival and overall survival rates. Notably, patients harboring both NOTCH1 mutations and lymph node metastasis exhibit a high recurrence rate, underscoring the mutation's prognostic significance.

**Conclusion**

The NOTCH1 C1133Y mutation exemplifies a gain-of-function alteration that disrupts normal receptor processing while activating oncogenic signaling pathways. Its role in promoting tumor progression and metastasis highlights its potential as a therapeutic target in OSCC. Further research into strategies that modulate NOTCH1 activity, possibly by enhancing FBXW7β-mediated degradation, could offer novel avenues for treatment.

For a comprehensive understanding, you may refer to the following key studies:

- Zheng Y, et al. "A novel Notch1 missense mutation (C1133Y) in the Abruptex domain exhibits enhanced proliferation and invasion in oral squamous cell carcinoma." Cancer Cell International, 2018. Same as 10.1
- Zheng Y, et al. "Isoform specific FBXW7 mediates NOTCH1 Abruptex mutation C1133Y deregulation in oral squamous cell carcinoma." Cell Death & Disease, 2020. Same as 10.1
- Song X, et al. "Common and complex Notch1 mutations in Chinese oral squamous cell carcinoma." PLoS ONE, 2013. Yes, article, 13

**dbGVOSCC output**

1. The information is: TP53P177L mutation is a likely pathogenic variant linked to oral squamous cell carcinoma (OSCC).


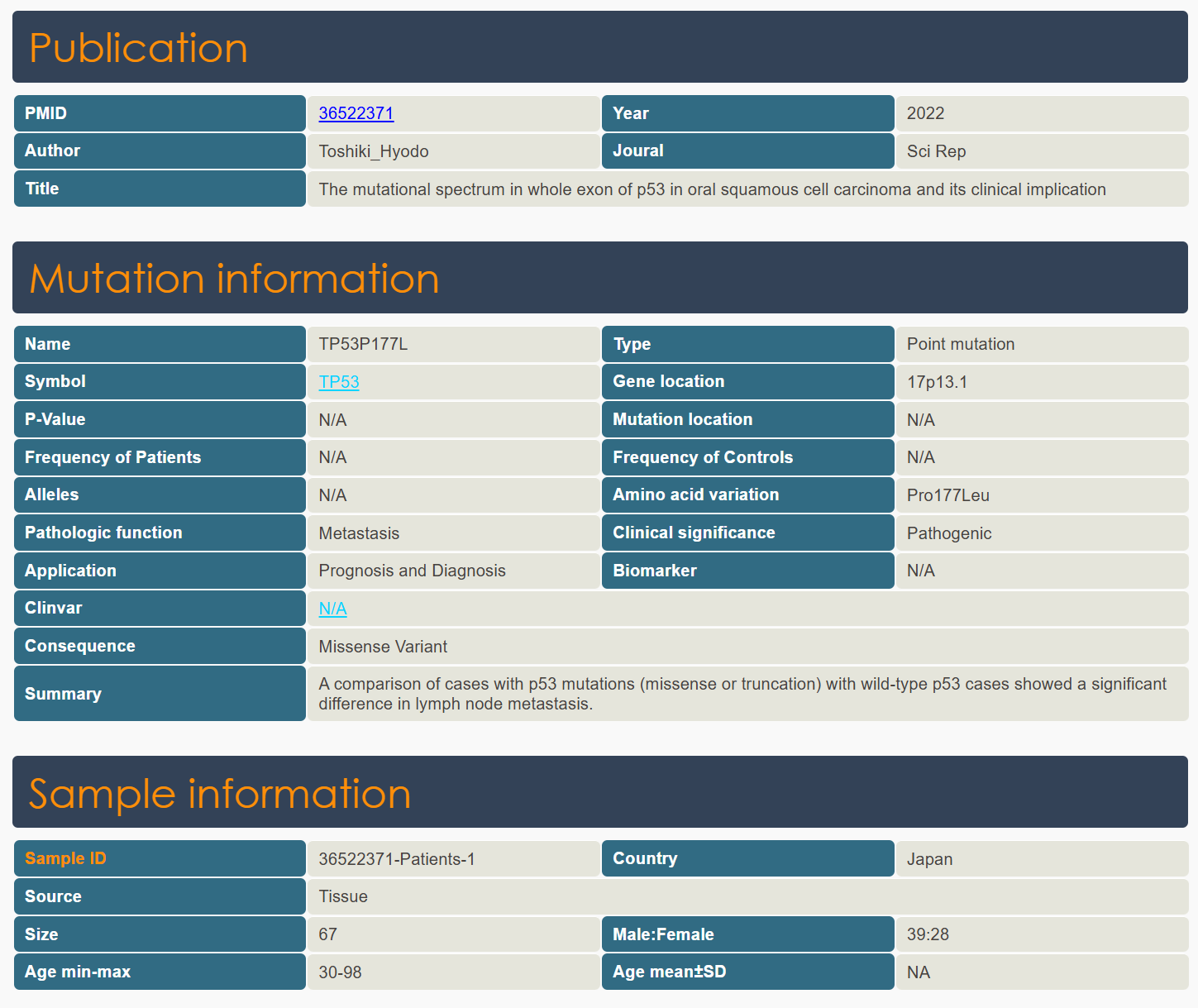


1. The information is: KRAS CNV mutation is a likely pathogenic variant linked to oral squamous cell carcinoma (OSCC).


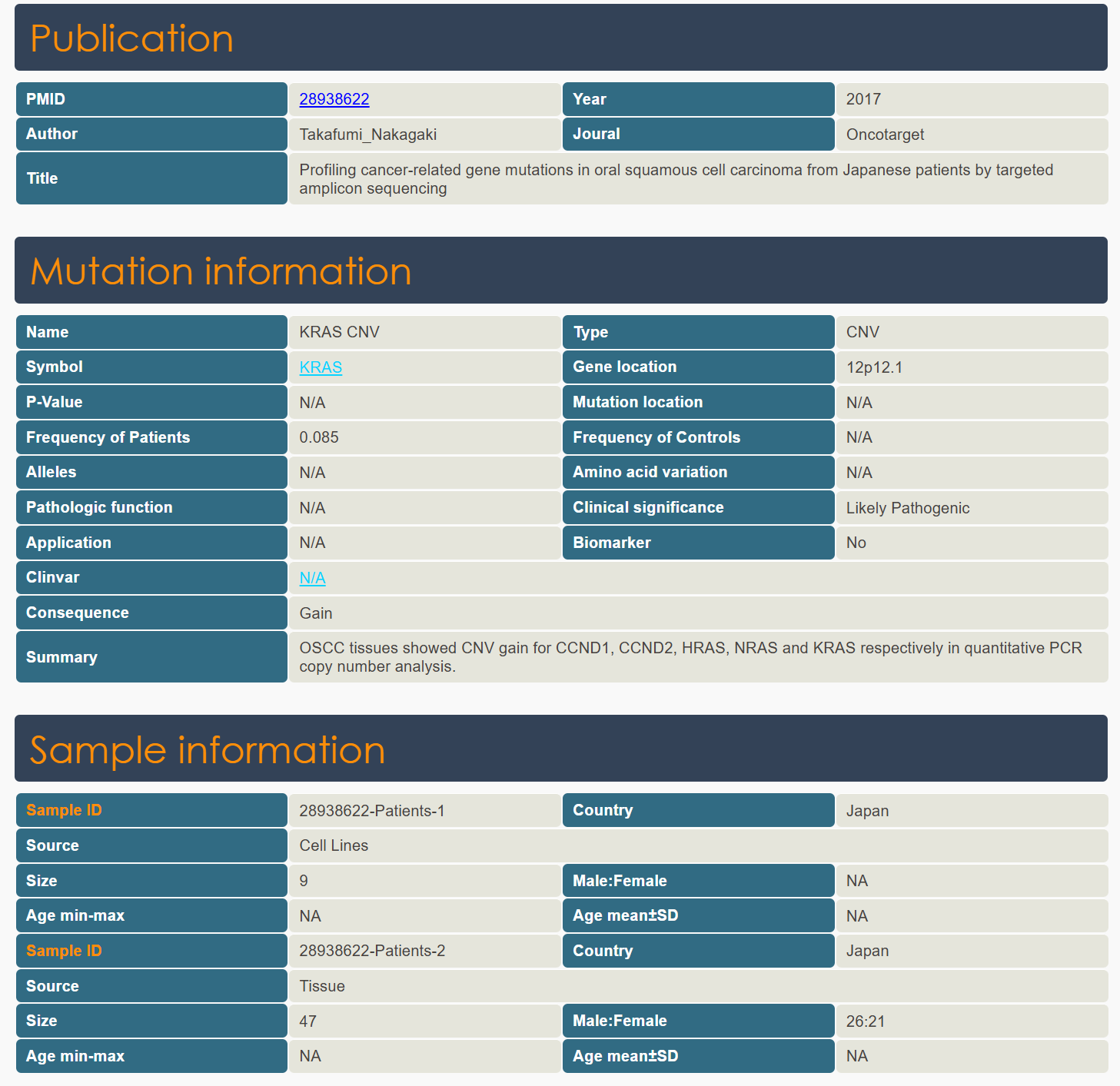


1. The information is: rs1138272 mutation is a likely pathogenic variant linked to oral squamous cell carcinoma (OSCC).


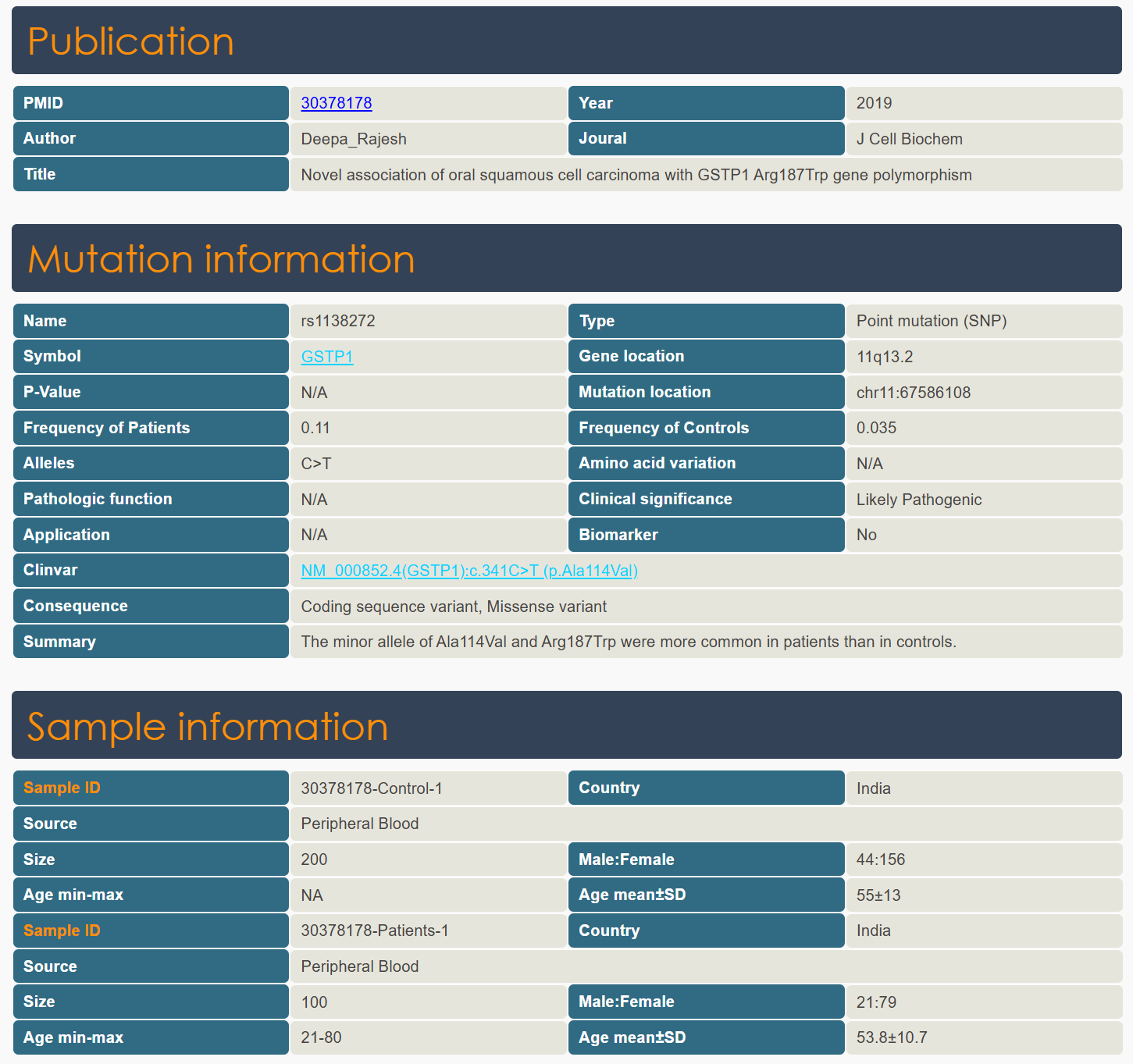


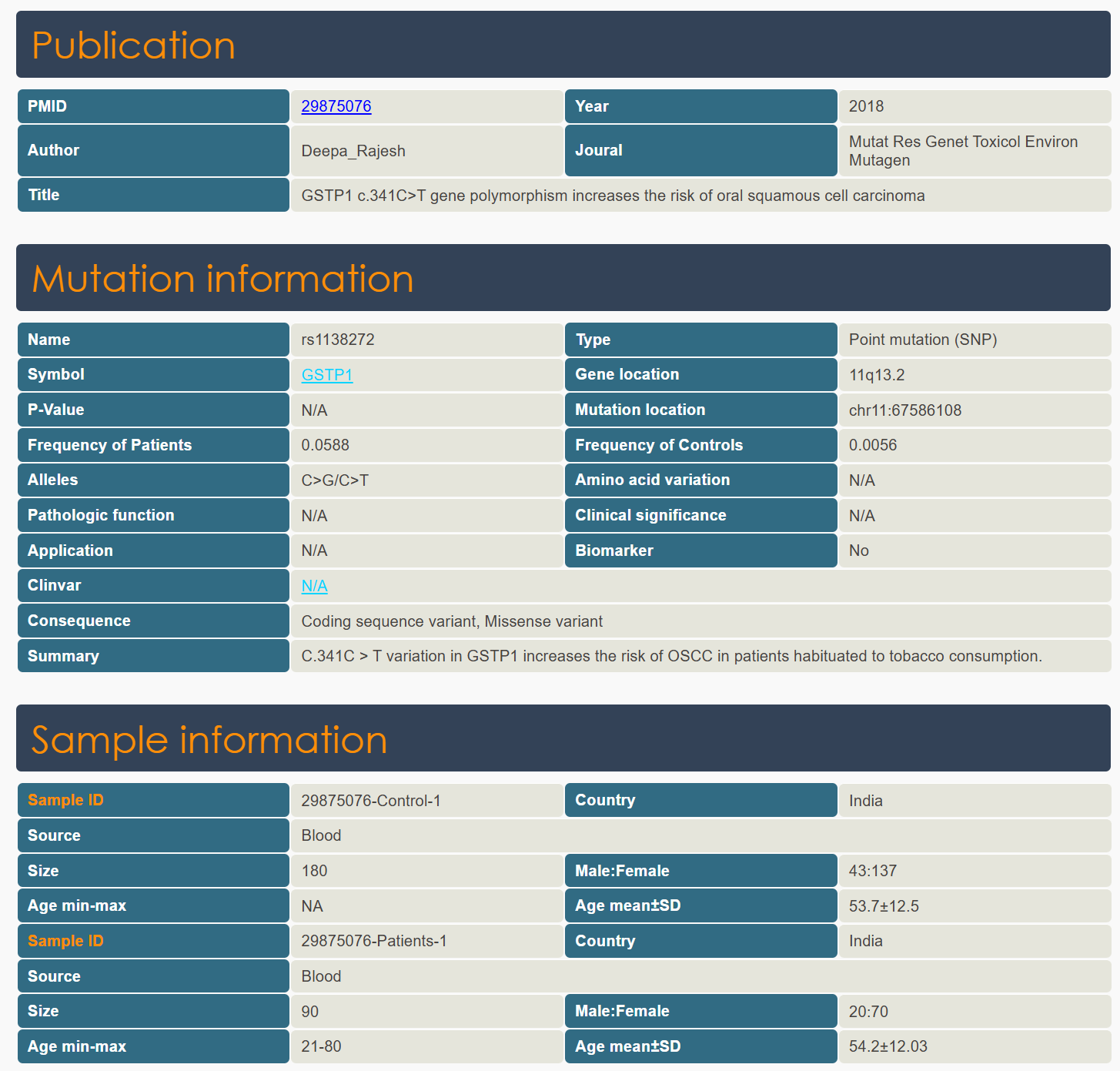


1. The information is: RB1CC1 homozygous deletion mutation is a likely pathogenic variant linked to oral squamous cell carcinoma (OSCC).


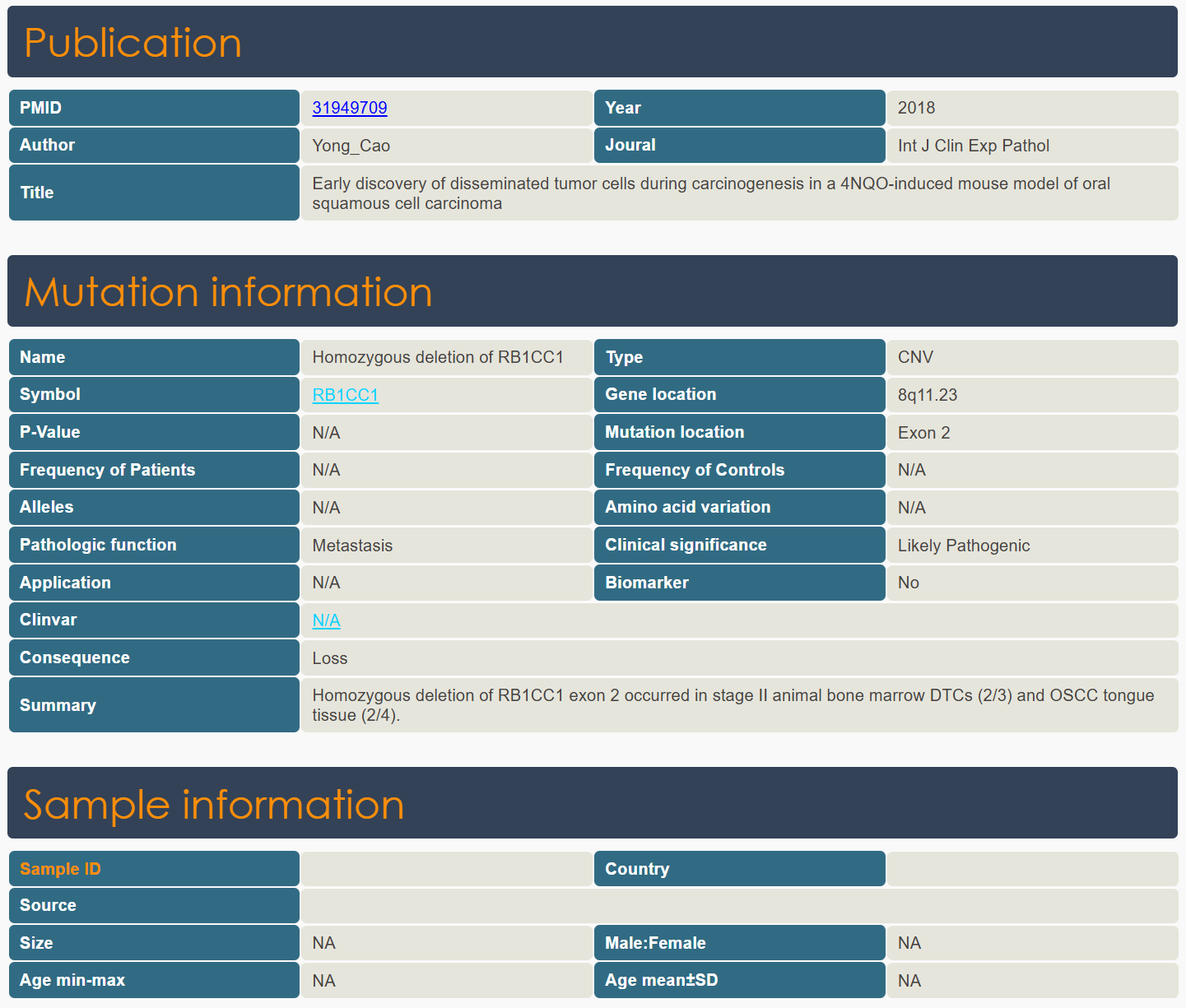


1. The information is: rs6950683 mutation is a likely benign variant linked to oral squamous cell carcinoma (OSCC).


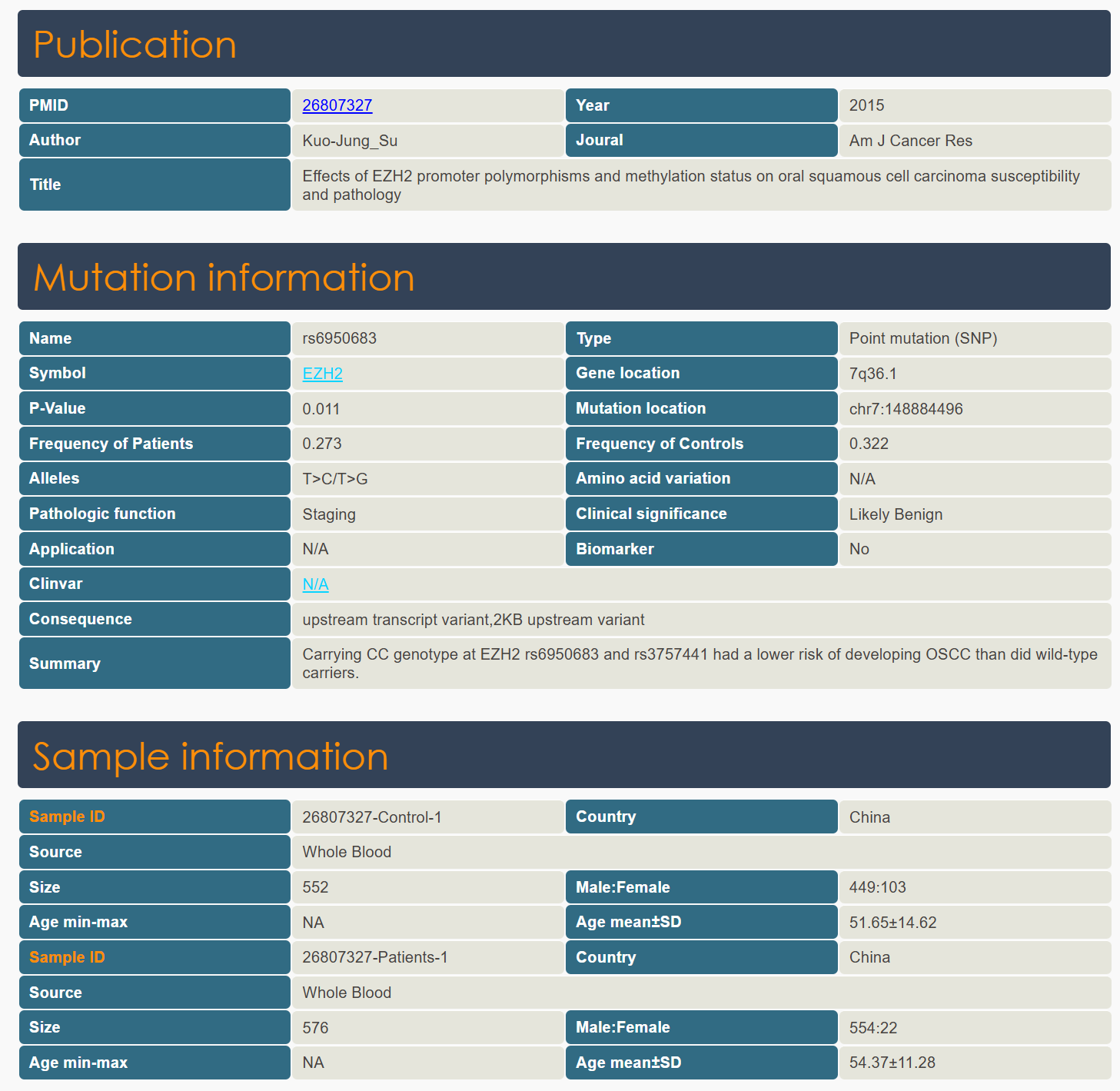


1. The information is: HIF-1a C1772T polymorphism mutation is a pathogenic variant linked to oral squamous cell carcinoma (OSCC).


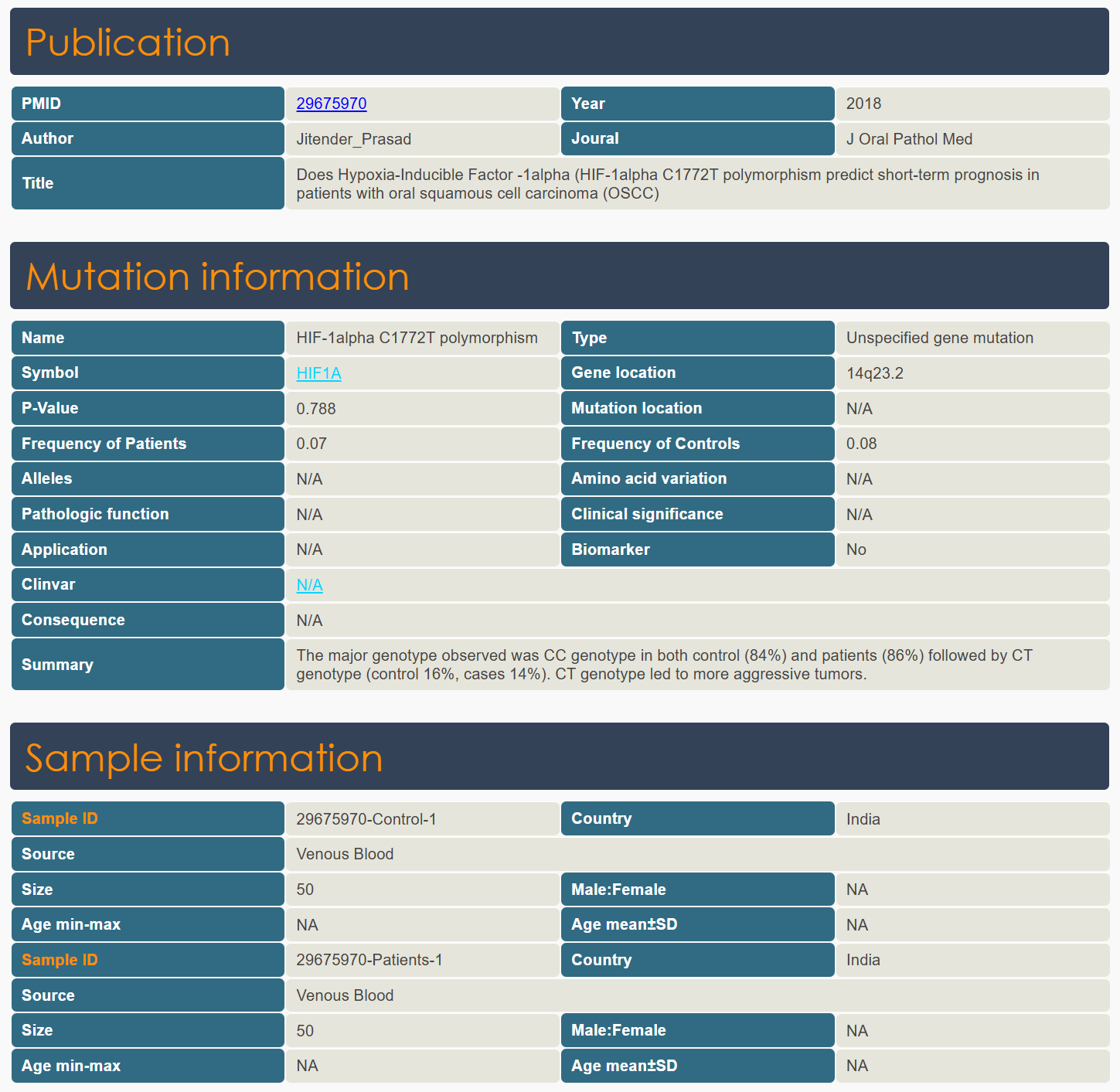


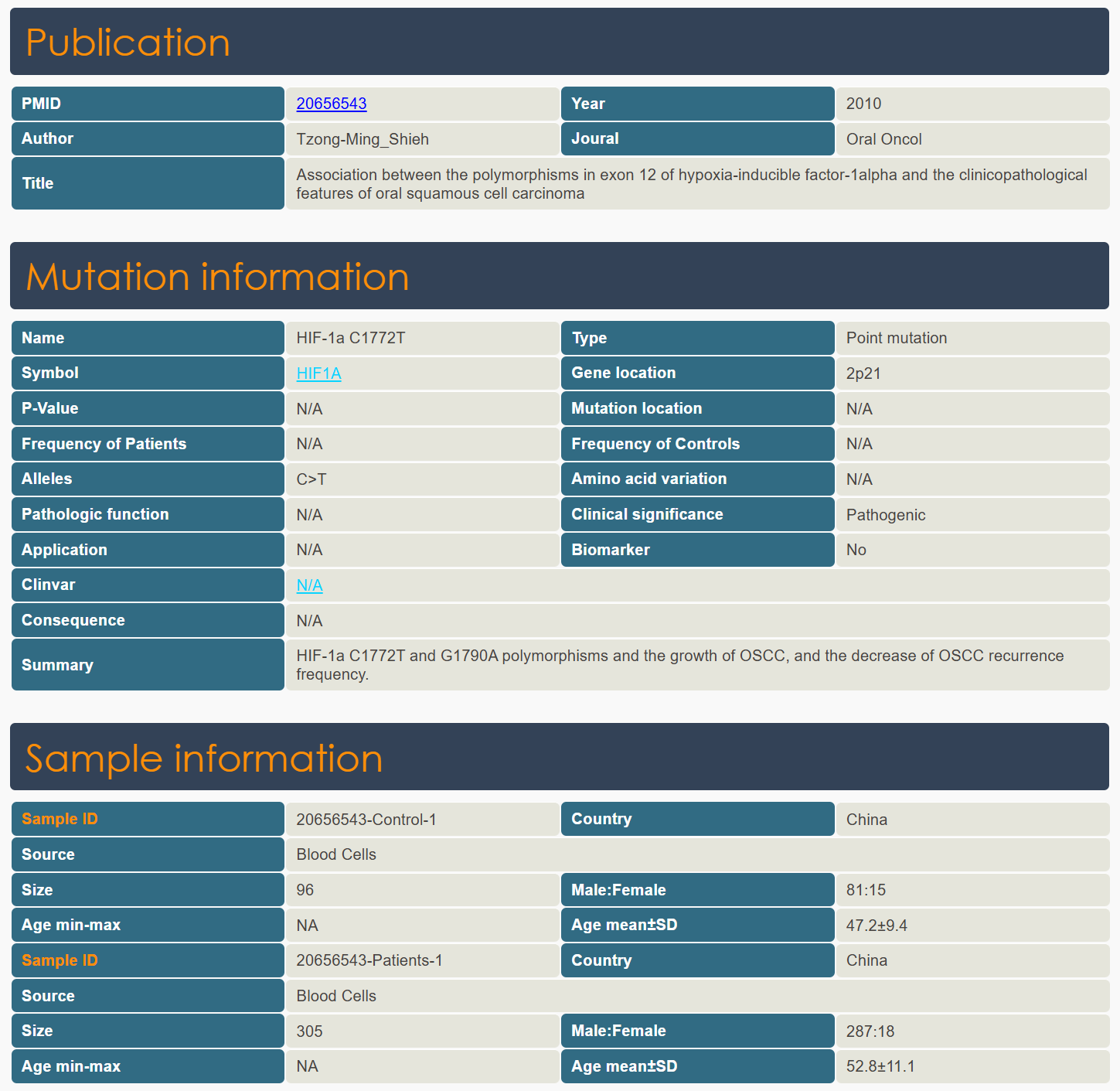


1. The information is: NOTCH1 c.842 mutation is a pathogenic variant linked to oral squamous cell carcinoma (OSCC).


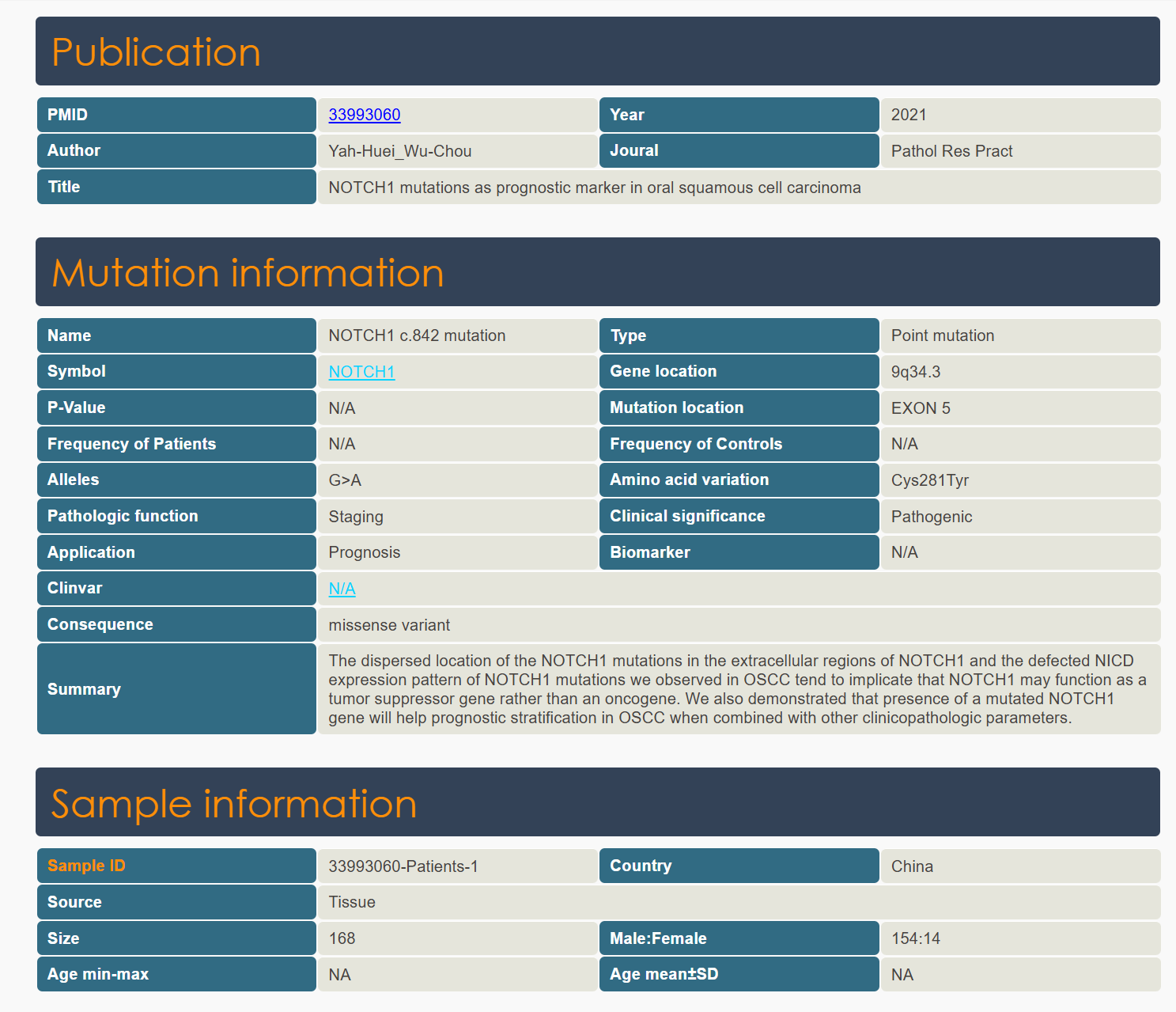


1. The information is: EGFR CA repeats polymorphism mutation is a pathogenic variant linked to oral squamous cell carcinoma (OSCC).


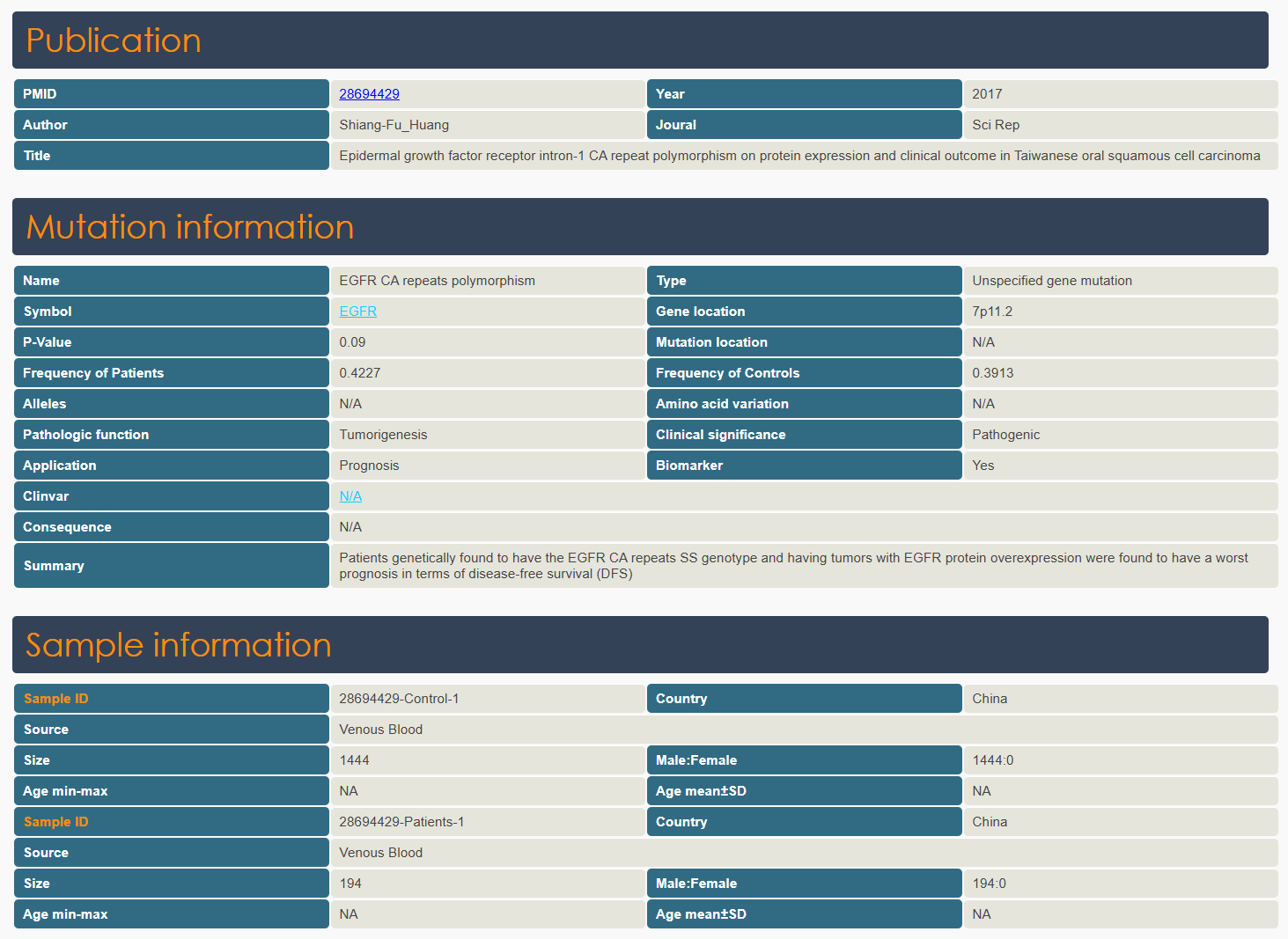


1. The information is: TGFBR2 receptor II mutation is a pathogenic variant linked to oral squamous cell carcinoma (OSCC).


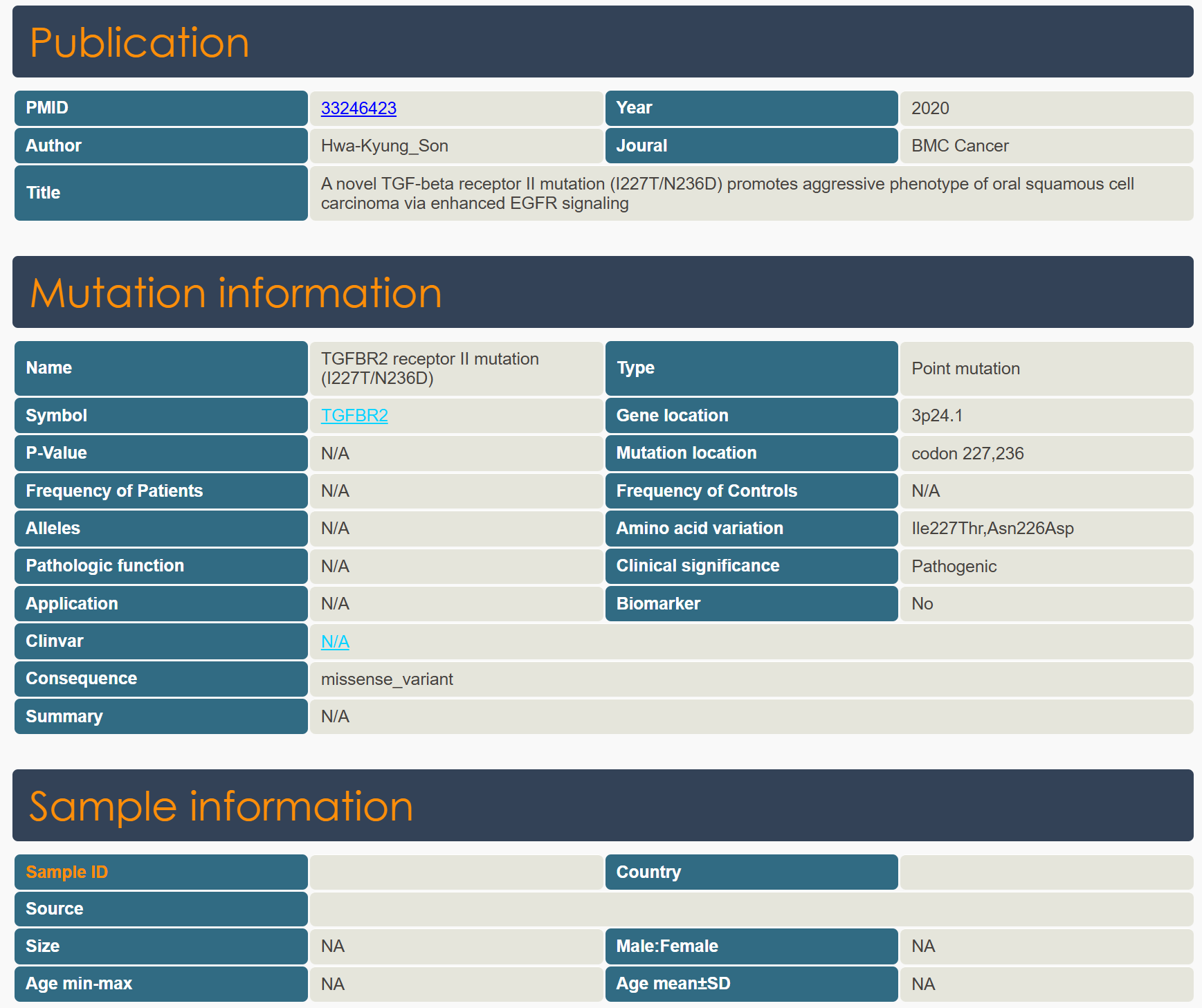


1. The information is: NOTCH1 C1133Y mutation is a pathogenic variant linked to oral squamous cell carcinoma (OSCC).


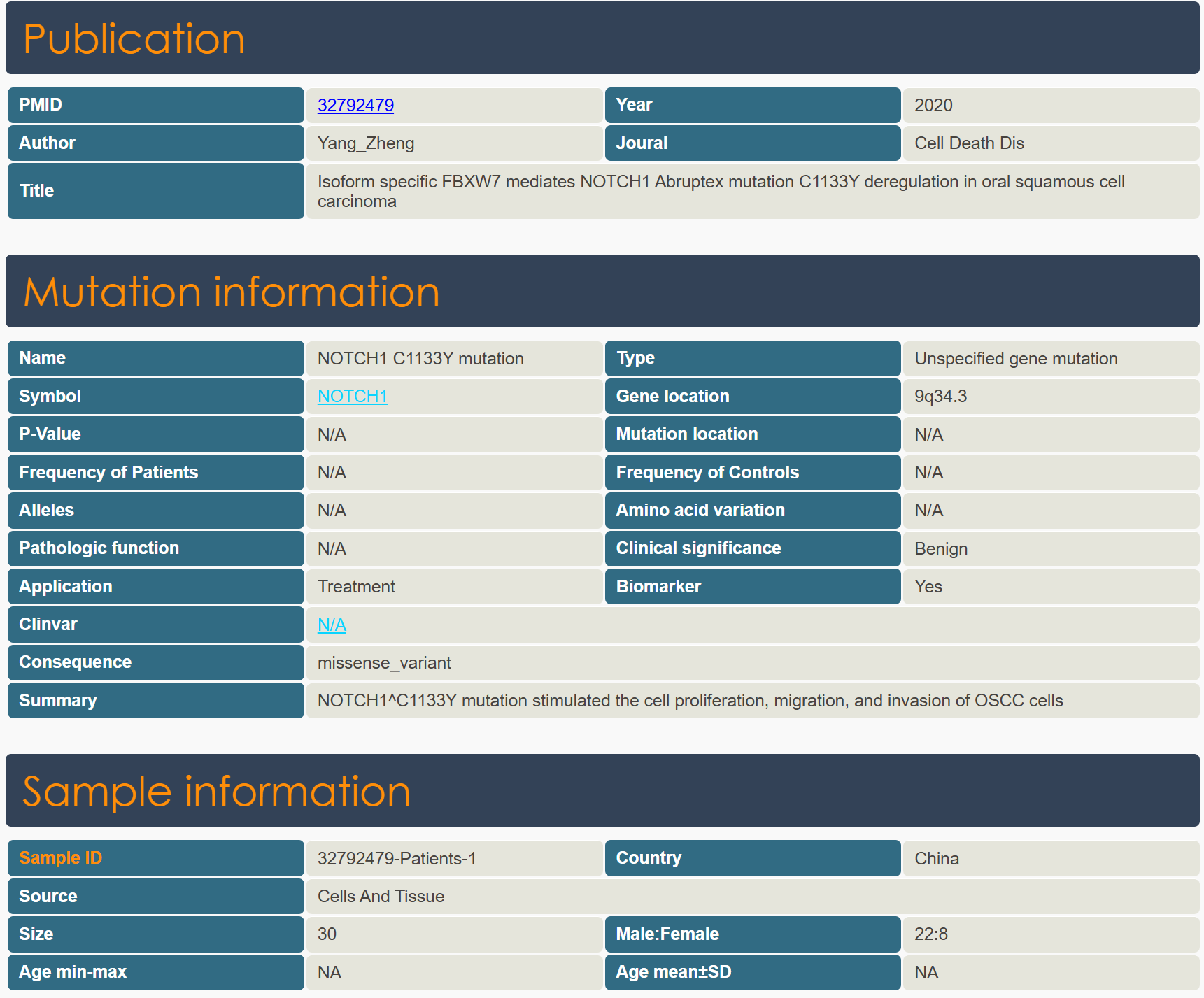


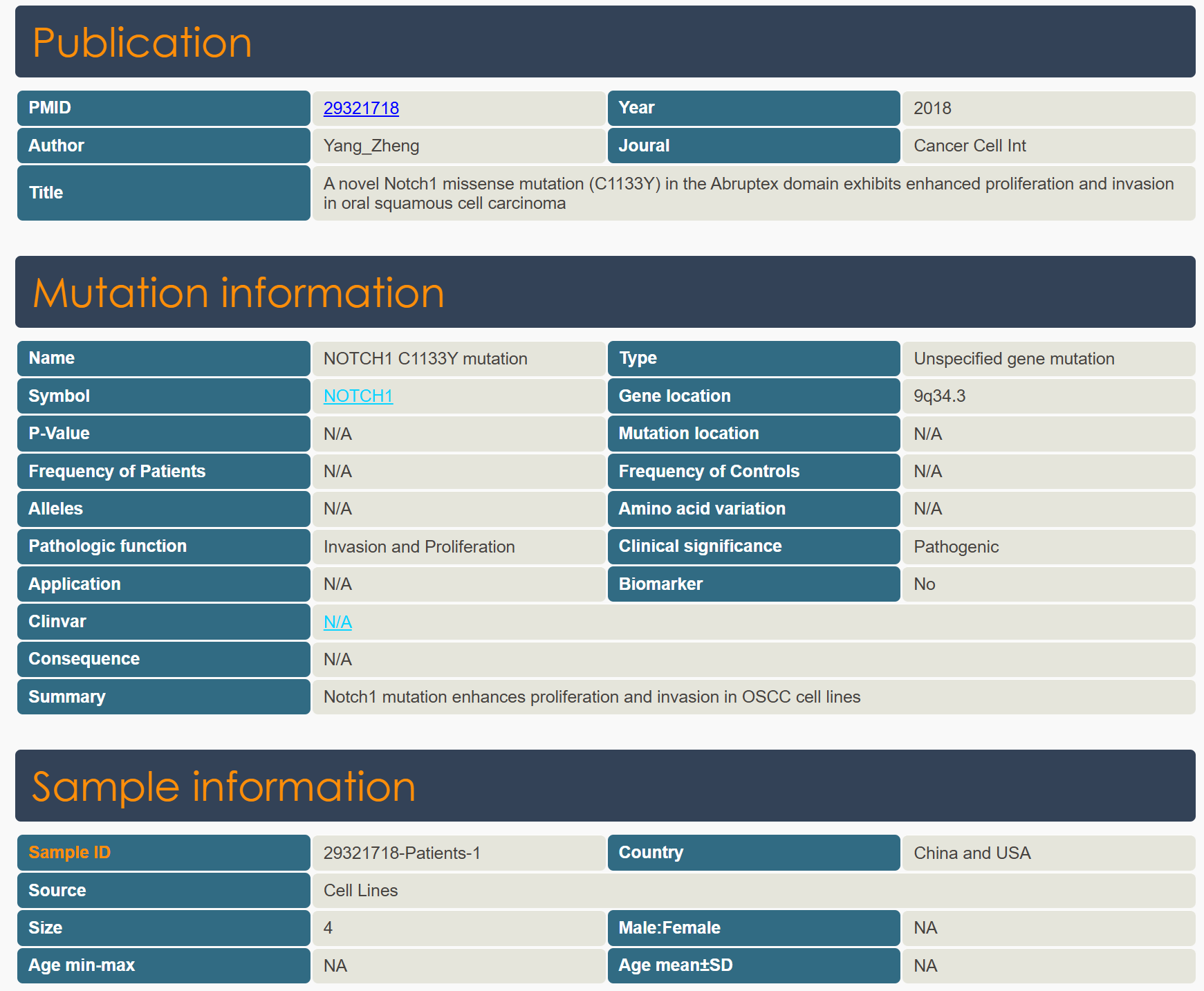

Supplement: Supplementary file 3 [file DataSheet1.docx]
